# Threonic acid, an ascorbic acid metabolite, synergizes with intermittent fasting to ameliorate obesity

**DOI:** 10.1038/s12276-025-01613-y

**Published:** 2026-01-09

**Authors:** Sungjoon Oh, Seokjae Park, Eun-Kyoung Kim

**Affiliations:** 1 Department of Brain Sciences, Daegu Gyeongbuk Institute of Science and Technology, Daegu, Republic of Korea; 2 Neurometabolomics Research Center, Daegu Gyeongbuk Institute of Science and Technology, Daegu, Republic of Korea

**Keywords:** Hypothalamus, Obesity

## Abstract

Intermittent fasting (IF) is a safe and sustainable approach for obesity treatment, yet its weight loss efficacy is relatively modest compared with that of pharmacologic anti-obesity therapies. The synergistic benefits of pairing IF with administration of nutrient-derived metabolites remain poorly understood. Here we report that combining IF with threonic acid (TA), an ascorbic acid metabolite, led to more pronounced reductions in body weight and food intake, as well as improvements in energy expenditure and glycemic control, compared with either intervention alone in diet-induced obese mice. These metabolic benefits were associated with the anorexigenic role of TA in reversing fasting-induced upregulation of the hypothalamic orexigenic neuropeptides NPY and AGRP. In the hypothalamus, TA competed with glucose for uptake via glucose transporter 3 (GLUT3), while IF boosted the TA uptake through both glucose depletion and upregulation of GLUT3, resulting in a more robust suppression of NPY and AGRP expression. Collectively, our findings highlight the combination of TA with IF as a promising metabolite-based combinatorial strategy to enhance the therapeutic efficacy of obesity treatment.

## Threonic acid boosts intermittent fasting for weight loss

Obesity is a growing health issue worldwide. Current treatments have side effects and limited long-term success. This study explores combining intermittent fasting (IF), a dietary approach involving alternating periods of eating and fasting, with either ascorbic acid (AA, also known as vitamin C) or its metabolite, threonic acid (TA), to enhance obesity treatment. In the study, mice were fed a high-fat diet to induce obesity and then subjected to IF and AA or TA treatments. Researchers found that IF combined with TA was more effective than IF with AA or either treatment alone in reducing body weight and improving metabolic health. These metabolic benefits were associated with TA’s appetite-suppressing action in reversing fasting-induced increases in hypothalamic orexigenic neuropeptides. The study concludes that TA could be a promising strategy for treating obesity.

This summary was initially drafted using artificial intelligence, then revised and fact-checked by the author.

## Introduction

The global prevalence of obesity and its associated complications continues to rise, posing a major public health concern^1,2^. Although pharmacologic anti-obesity therapies including recent advances in gut-hormone-based medications offer therapeutic benefits, their long-term efficacy remains limited and their use is often accompanied by adverse effects^3,4^. These limitations have spurred growing interest in alternative interventions, notably intermittent fasting (IF), a dietary regimen characterized by alternating periods of fasting and eating^5–7^. Various IF models including time-restricted eating, periodic fasting, fasting-mimicking diets and alternate-day fasting demonstrate consistent benefits in obesity management^5–7^. In both humans and rodent models of obesity, IF induces robust weight loss and metabolic improvements by reducing pro-inflammatory cytokine production and immune cell activity, enhancing fat oxidation, lipolysis and browning of white adipose tissue, and improving glucose tolerance and insulin sensitivity^8–15^.

Despite its potential as a safe and sustainable anti-obesity strategy, the long-term efficacy of IF for weight reduction remains modest compared with pharmacological therapies^16,17^. Clinical studies indicate that patients with obesity typically lose only about 0.25 kg per week or 3% of body weight over 3 months^16,17^. Given the complex pathogenesis of obesity involving an interplay among genetic, environmental and hormonal factors, monotherapy with IF may have limited efficacy in achieving substantial obesity reduction^18^. Consequently, combination therapies incorporating multiple interventions with complementary mechanisms of action are increasingly recommended for more effective obesity treatment^19,20^. Recent evidence indicates that combining IF with behavior-based interventions such as exercise training can enhance weight loss in both humans and rodent models of obesity^21,22^. However, the potential synergistic effects of integrating IF with other interventions for obesity treatment remain largely unexplored.

Ascorbic acid (AA), also known as vitamin C, is a water-soluble vitamin widely consumed as a dietary supplement^23^. AA has garnered attention as a potential anti-obesity therapy, due to its antioxidant and anti-inflammatory properties in peripheral tissues including adipose tissue, liver, pancreas and endothelium^24–32^. AA exerts various metabolic benefits such as reducing inflammatory mediators^24–26^, improving lipid metabolism by decreasing cholesterol synthesis and absorption while promoting lipolysis in adipocytes^27–29^, and enhancing whole-body glucose disposal and nonoxidative glucose metabolism^30–32^. However, AA’s therapeutic potential is often limited by its inherent instability and suboptimal pharmacokinetic profile, as it rapidly oxidizes in oxygen- and light-rich environments^33^. AA is sequentially metabolized to dehydroascorbic acid (DHA), 2,3-diketogulonic acid (DKGA), oxalic acid (OA) and threonic acid (TA)^34^. These metabolites, particularly DHA, may reduce oxidative stress and inflammation^35,36^. Despite these promising properties, the anti-obesity potential of AA and its metabolites, especially in combination with IF, has not been investigated.

Interestingly, AA is more abundant in the human brain than in other organs^37^. Within the brain, the hypothalamus has particularly high AA levels (up to 229 mg/kg of tissue); the import of AA is facilitated by a high-affinity AA transporter in hypothalamic neurons^38–40^. The hypothalamus, a central brain region that controls whole-body energy homeostasis, contains the arcuate nucleus (ARC), which houses two key appetite-regulating neuronal populations: one expressing the orexigenic neuropeptides neuropeptide Y (NPY) and agouti-related peptide (AGRP), and the other expressing the anorexigenic neuropeptides proopiomelanocortin (POMC) and cocaine- and amphetamine-regulated transcript (CART)^41,42^. These neurons play a pivotal role in energy homeostasis by modulating body weight, appetite, energy expenditure (EE), glucose homeostasis and brown adipose tissue (BAT) thermogenesis^41–44^. Intriguingly, vitamin D reduces body weight by decreasing food intake through the activation of POMC neurons^45^. However, the dynamics of AA metabolism in the hypothalamus and its roles in the hypothalamic regulation of energy homeostasis remain unknown.

In this study, we demonstrated that combining IF with TA is more effective in improving metabolic parameters such as body weight, food intake, EE and glycemic control in diet-induced obese (DIO) mice than combining IF with AA or either intervention alone. Furthermore, we explored the mechanisms underlying the synergistic effects of these combination therapies.

## Materials and methods

### Chemicals and reagents

Sodium ascorbate (Sigma-Aldrich, 1613509), DHA (Sigma-Aldrich, 261556), DKGA (Pharmaaffiliates, 27 00698), OA (Sigma-Aldrich, 41706) and TA hemicalcium salt (Sigma-Aldrich, 380644) were dissolved in phosphate-buffered saline (PBS) for use with N41 cells and in saline for use in mice; PBS and saline were used as a vehicle (Veh) in respective experiments. ^13^C-labeled AA ([^13^C_6_]-AA; Sigma-Aldrich, 795097) and glucose ([^13^C_2_]-glucose; Sigma-Aldrich, 453188) were dissolved in PBS for use with N41 cells and in saline for use in mice. For measuring reactive oxygen species (ROS), glutathione reduced ethyl ester (GSH-MEE; Sigma-Aldrich, G1404) and hydrogen peroxide (H_2_O_2_; Sigma-Aldrich, H1009) were dissolved in PBS. For pharmacological inhibition studies, sulfinpyrazone (Sigma-Aldrich, PHR3244) and KL-11743 (Sigma-Aldrich, SML3458) were dissolved in dimethyl sulfoxide (Sigma-Aldrich, D2650). For liquid chromatography–tandem mass spectrometry (LC–MS/MS) analysis, high-performance liquid chromatography (HPLC)-grade water (W6), methanol (A456) and acetonitrile (A996) were purchased from Thermo Fisher Scientific and formic acid (Supelco 5.33002) was purchased from Merck. In all experiments, neutral pH was maintained.

### Animal studies

C57BL/6N male and female mice at 7 weeks of age were purchased from Koatech. NPY-hrGFP mice (JAX#006417), which express humanized Renilla green fluorescent protein in hypothalamic NPY neurons under the control of the mouse *Npy* promoter, were provided by Dr. Ki-Woo Kim (Yonsei University). All mice were housed in a specific pathogen-free animal facility in individually ventilated cages under controlled conditions (23 ± 3 °C, 50% ± 10% humidity) with a 12-h light/dark cycle (light: 07:00 to 19:00; dark: 19:00 to 07:00). Mice were fed a high-fat diet (HFD, 60% kcal from fat; Envigo, 06414) for 16 weeks to generate DIO mice, followed by either ad libitum (AL) or IF regimens on the same HFD for 50 days. The IF regimen follows an alternate-day fasting pattern involving 24-h fasting followed by 24-h feeding. Food intake and body weight were measured daily, and mice had free access to water throughout the experiment. Metabolic measurements including body composition, indirect calorimetry and BAT thermogenesis were performed on a day when all mice were in a fed state. This ensured that fasted mice were assessed under the same nutritional conditions as the AL controls. All animal studies followed the National Institutes of Health guidelines and were approved by the Institutional Animal Care and Use Committee at the Daegu Gyeongbuk Institute of Science and Technology Laboratory Animal Resource Center (approval number DGIST-IACUC-24062705-0000).

### Intraperitoneal injections of AA, [^13^C_6_]-AA or TA

DIO mice received daily intraperitoneal (i.p.) injections of Veh (saline), AA (up to a maximum of 15 mmol/kg) or TA (up to a maximum of 1.5 mmol/kg) under AL or IF. Injections were administered before the onset of the dark cycle (7:00 pm). To trace AA metabolism in the hypothalamus, we made a single i.p. injection of [^13^C_6_]-AA (0.3 mmol/kg) mixed with unlabeled AA (14.7 mmol/kg) at a 1:50 ratio to fed or 24-h-fasted DIO mice.

### Body composition and indirect calorimetry

After 50 days of Veh, AA or TA injections, mice were anesthetized with an i.p. injection of Zoletil 50 (30 mg/kg body weight; Virbac) and Rompun (10 mg/kg body weight; Bayer Korea) in saline. Body composition was then assessed using iNSiGHT DXA (Osteosys). Following these measurements, mice were individually housed in metabolic chambers of the Comprehensive Lab Animal Monitoring System (Columbus Instruments). Daily Veh, AA or TA injections were continued under the respective feeding regimens (AL or IF), and metabolic parameters were recorded for 24 h after the second injection. Oxygen consumption (VO_2_), carbon dioxide production (VCO_2_), heat production and locomotor activity were determined using an Oxymax System (Columbus Instruments). The respiratory exchange ratio (RER) was calculated as VCO_2_/VO_2_. Locomotor activity was assessed by measuring interruptions in infrared beams along the *X* and *Z* axes (total ambulatory counts). Regression-based analysis of EE was performed using analysis of covariance (ANCOVA), with heat production as the dependent variable and lean body mass as a covariate, as previously described^46,47^.

### BAT thermogenesis

The surface temperature of interscapular BAT in nonanesthetized DIO mice was measured on day 50 after the final i.p. injection of Veh, AA or TA using a FLIR infrared camera (FLIR Systems, E6). Infrared images of mice were captured from a 30-cm distance, and temperature data were analyzed in FLIR analysis software.

### Glucose and insulin tolerance tests

Glucose and insulin tolerance tests were performed on day 50 after the final i.p. injection of Veh, AA or TA. Mice were fasted for 16 h before the glucose tolerance test and 6 h before the insulin tolerance test. Following an i.p. injection of glucose (2 g/kg; Sigma-Aldrich, G7021) or human insulin (1 U/kg; Sigma-Aldrich, I9278), blood glucose levels were monitored over a 2-h period via tail vein sampling using an Accu-Check II glucometer (Roche).

### Hypothalamic cell line culture

The embryonic mouse hypothalamic mHypoE-N41 (CLU121) cell line (hereafter referred to as N41) was purchased from Cellutions Biosystems and cultured in Dulbecco’s modified Eagle medium (DMEM; Sigma-Aldrich, D5796) supplemented with 10% fetal bovine serum (FBS; Hyclone Laboratories Inc., SH30910.03) and 1% penicillin–streptomycin (Hyclone Laboratories, SH30236.01) at 37 °C. The cells were screened for mycoplasma contamination by Cellutions Biosystems. To induce glucose deprivation, standard DMEM containing 25 mM glucose was replaced with glucose-free DMEM (Welgene, LM001-56) supplemented with 10% FBS and 1% penicillin–streptomycin, and cells were maintained in this medium for 4 h.

### Cell viability assay

Cell viability was assessed through the CellTiter-Blue assay (Promega, G8080), which is based on measuring the reduction of resazurin to resorufin by metabolically active cells. Cells were grown to confluence in 96-well plates in the presence or absence (control) of individual chemicals. After 24-h incubation at 37 °C, 20 µl of CellTiter-Blue reagent was added to each well and incubated for 4 h at 37 °C. Resorufin production was quantified by measuring absorbance at 590 nm in a MicroQuant Plate Reader (Bio-Tek Instruments) and was expressed as a percentage relative to control cells.

### Gene KD by siRNA transfection

ON-TARGETplus small interfering RNAs (siRNAs) for control (D-001810-10-20), *Svct2* (L-062312-01-0010), *Glut1* (L-044254-01-0010) and *Glut3* (L-059103-01-0010) were purchased from Dharmacon. Cells were transfected with 100 nM siRNAs for 48 h using Lipofectamine 3000 (Invitrogen). Knockdown (KD) efficiency was confirmed by western blotting or quantitative real-time polymerase chain reaction (PCR).

### ROS measurement

Intracellular ROS levels were measured using the ROS-sensitive probe 2′,7′-dichlorodihydrofluorescein diacetate (CM-H_2_DCFDA, Invitrogen, C6827). CM-H_2_DCFDA was dissolved in dimethyl sulfoxide to prepare a 10 mM stock solution, which was further diluted before use. Cells were collected using 0.05% trypsin–EDTA solution, stained with CM-H_2_DCFDA (200 nM) for 30 min at 37 °C in the dark and immediately analyzed using an Accuri C6 flow cytometer (BD Biosciences). Nonstained cells served as a negative control, and cells treated with 10 mM H_2_O_2_ were used as a positive control.

### Western blot analysis

Samples (hypothalamic tissue and N41 cells) were lysed as previously described^48^. Protein concentrations were determined using a BCA assay kit (Thermo Fisher Scientific, 23228). Equal amounts of protein (12 µg per lane) were separated by SDS–PAGE and transferred onto an Immobilon-P membrane (Merck, IPVH00010). The membrane was blocked with 5% skim milk in TBST for 1 h and was then incubated with the primary antibody for 1 h at room temperature (RT) or overnight at 4 °C. After washing, the membrane was incubated with a horseradish-peroxidase-conjugated secondary antibody for 1 h at RT. Protein bands were detected using the SuperSignal West Pico Chemiluminescent Substrate (Thermo Fisher Scientific, 34580), and signal intensity was quantified with ImageJ software (National Institutes of Health). The antibodies are listed in Supplementary Table 1.

### Quantitative real-time PCR

Samples (hypothalamic tissue, N41 cells and BAT) were prepared as previously reported^48^. Total RNA was isolated using TRIzol reagent (Invitrogen, 15596018), and the RNA pellet was dissolved in nuclease-free water (Promega, P1195). RNA concentration was measured using a NanoDrop spectrophotometer (Thermo Fisher Scientific). cDNA was synthesized from 3 µg of RNA using GoScript Reverse Transcriptase (Promega, A5001). mRNA levels were quantified by quantitative real-time PCR using TB Green (SYBR) Premix Ex Taq II (TaKaRa, RR820L). *Actb* was used as the reference gene for normalization. Experiments with N41 cells and mouse tissues were performed using at least three biological replicates, each with an average of three technical replicates. Primer sequences are listed in Supplementary Table 2.

### LC–MS/MS analysis

For the analysis of both ^13^C-labeled and unlabeled AA metabolites and glucose, N41 cells were extracted with 200 µl of 80% methanol in water containing 0.1% formic acid, while hypothalamic tissue was extracted with 300 µl of 80% acetonitrile in water containing 0.1% formic acid and homogenized using a MagNA Lyser (Roche). Each lysate was centrifuged at 16,000*g* for 15 min, and the supernatant was filtered through a 0.2-µm syringe filter. To prevent AA oxidation, all procedures were conducted under reduced light (using amber tubes), with minimal oxygen exposure at 4 °C and acidic pH. LC–MS/MS analysis was performed using an Agilent 1290 ultraperformance liquid chromatography system coupled to an Agilent 6490 triple quadrupole mass spectrometer. Samples (2 µl) were separated on a Phenomenex Synergi HydroRP column (150 mm × 2 mm, 4 µm, 80 Å) maintained at 45 °C. Mobile phase A consisted of 10 mM ammonium acetate and 0.1% formic acid in water, and mobile phase B was acetonitrile. The flow rate was 0.3 ml/min, and the total run time was 9 min. The gradient was as follows: 0–2 min at 100% A; 2–5 min, linear gradient to 90% B; 5–7 min, 90% B; 7–7.1 min, return to initial conditions; and 7.1–9 min, re-equilibration. The system operated in multiple-reaction monitoring mode using optimized collision energy in negative electrospray ionization mode. Selection of precursor-to-fragment ion transitions was based on previous reports^49,50^, and ^13^C-labeled AA metabolites and ^13^C-labeled glucose were identified according to our mass spectrometry settings (Supplementary Table 3).

### Immunohistochemistry

Samples were prepared and staining intensity was quantified as previously reported^48^. In brief, anesthetized NPY-hrGFP DIO mice were perfused with PBS, followed by 4% paraformaldehyde fixation. The brains were extracted and postfixed in 4% paraformaldehyde for 16 h at 4 °C and then transferred to 30% sucrose in PBS. The brains were embedded in Optimal Cutting Temperature compound (Leica, 3801480), frozen on dry ice and stored at −80 °C. The brains were sliced (thickness 35 µm) and blocked in 5% normal donkey serum with 1% bovine serum albumin for 1 h at RT. Brain slices were incubated overnight at 4 °C with the primary antibody in blocking buffer. After washing, they were incubated with secondary antibody (Cy3 conjugated to detect GLUT3; 1:500) in blocking buffer for 2 h at RT and washed. Fluorescence images were taken with a Carl Zeiss LSM 800 confocal laser-scanning microscope and analyzed in Zen software (Carl Zeiss). Quantification of GFP-positive cell numbers and GLUT3 fluorescence intensity within GFP-positive cells was performed using Fiji of ImageJ 2 software (National Institutes of Health) on eight brain slices from each of four mice. The antibodies are listed in Supplementary Table 1.

### Statistical analysis and drawing chemical structures

GraphPad Prism software (v.10.0.2) was used for data visualization and statistical analysis, and ChemDraw software (v.20.0) was used to draw chemical structures. Statistical comparisons between two groups were performed using a two-tailed unpaired Student’s *t*-test. For multiple group comparisons, two-way analysis of variance (ANOVA) followed by Tukey post-hoc tests was used to determine the statistical significance of differences. Differences in EE were evaluated using ANCOVA, with body weight as a covariate. Quantitative data were presented as mean ± standard error of the mean (s.e.m.), and statistical significance was defined as *P* < 0.05.

## Results

### Combining IF with AA enhances its anti-obesity effects in DIO mice

**Fig. 1:**
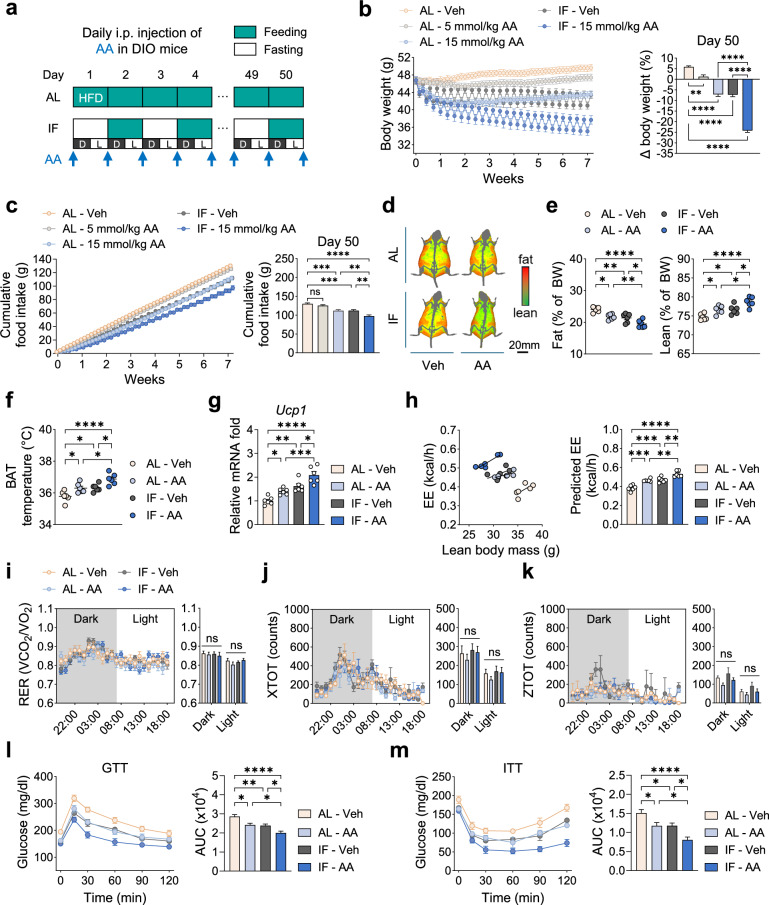
Combining AA with IF enhances anti-obesity effects in DIO mice. **a** Design of i.p. injections in DIO mice under AL or IF regimens. **b**, **c** Changes in body weight measured daily (left) and on day 50 (relative to day 0, right) (**b**) and cumulative food intake measured daily (left) and on day 50 (relative to day 0, right) (**c**) in male mice injected intraperitoneally with AA (*n* = 6). **d** Representative images of body composition. Red, fat mass; green, lean mass. **e** Fat mass (left) and lean mass (right) on day 50 (*n* = 6). **f** Interscapular BAT temperature (*n* = 6). **g** Relative mRNA levels of *Ucp1* in interscapular BAT (*n* = 6). **h** Left: regression-based analysis of EE against lean body mass. Right: bar graph indicating EE values adjusted for lean body mass using ANCOVA (*n* = 6). **i**–**k** Indirect calorimetry parameters: RER (**i**), locomotor activity (*X* axis) (**j**) and locomotor activity (*Z* axis) (**k**) (*n* = 6). **l** Glucose tolerance test (GTT) (*n* = 6). **m** Insulin tolerance test (ITT) (*n* = 6). Statistical significance was determined by two-way ANOVA followed by a post-hoc Tukey test. *P < 0.05, **P < 0.01, ***P < 0.001, ****P < 0.0001; ns no significance, AUC area under the curve, D dark phase, L light phase. Data are mean ± s.e.m.

To investigate whether the combination of IF and AA synergistically ameliorates obesity, we first assessed the weight loss efficacy of a single AA administration in male DIO mice fed an HFD for 16 weeks under AL feeding conditions using dose-ranging studies. Within the established safe range for i.p. AA administration (<23 mmol/kg)^51^, 15 mmol/kg AA produced the most significant reduction in body weight (Supplementary Fig. 1a) and raised plasma AA concentrations to 18.2 mM within 2 h, followed by a gradual decline consistent with reported pharmacokinetics^52^ (Supplementary Fig. 1b). Given that most of the administered AA was rapidly cleared from plasma within 12 h, we adopted a daily repeat dosing strategy to maintain systemic exposure. Previous preclinical obesity studies involving pharmacological agents such as liraglutide, which also utilized daily dosing, demonstrated that maximal weight loss typically occurs within the first 50 days before reaching a plateau^53–55^. Accordingly, we implemented prolonged daily dosing regimens (up to 50 days) to enable a comprehensive evaluation of metabolic adaptation, sustained anti-obesity efficacy and potential safety outcomes. Male DIO mice were assigned to either AL or IF regimen for 50 days, with daily i.p. injections of Veh or AA (Fig. 1a). Metabolic parameters of DIO mice were compared across the Veh and AA-treated groups under AL and IF conditions: AL-Veh, AL-AA (5 or 15 mmol/kg), IF-Veh and IF-AA (15 mmol/kg). No signs of sickness were observed over the 50-day injection period.

Under AL feeding, AA reduced body weight and food intake in a dose-dependent manner (Fig. 1b, c). When combined with IF, AA led to a greater reduction in body weight (30.1%) and food intake (25.2%) on day 50 compared with AL controls than did IF or AA alone (Fig. 1b, c). Moreover, IF combined with AA significantly reduced fat mass, increased interscapular surface temperature and elevated *Ucp1* mRNA levels in BAT compared with IF or AA alone (Fig. 1d–g). Indirect calorimetry revealed that IF combined with AA increased EE compared with IF or AA alone, while RER and locomotor activity remained unchanged across all groups (Fig. 1h–k). Furthermore, glucose tolerance and insulin sensitivity were significantly improved by IF combined with AA compared with IF or AA alone (Fig. 1l, m). Collectively, these findings demonstrate that the combination of IF and AA enhances the anti-obesity effects in male DIO mice in comparison with either intervention alone.

To examine the anti-obesity effects of IF combined with AA in female DIO mice, we followed the same experimental design (Fig. 1a). Similar to males, females receiving the combination therapy had greater reductions in body weight (27.5%) and food intake (23.0%) than those receiving either intervention alone, along with elevated EE (Supplementary Fig. 2). Thus, subsequent experiments were conducted in male DIO mice.

### Fasting increases AA uptake in the hypothalamus via SVCT2, leading to the downregulation of orexigenic neuropeptide expression

**Fig. 2:**
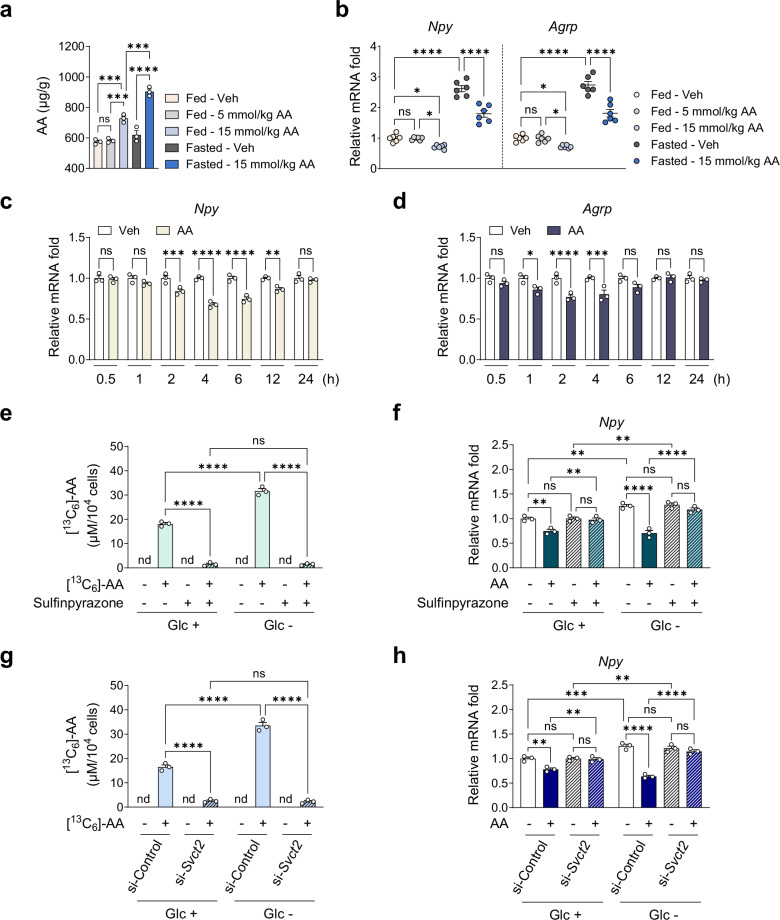
Fasting increases SVCT2-mediated AA uptake in the hypothalamus, downregulating the expression of orexigenic neuropeptides. **a**, **b** Male DIO mice fed a HFD for 16 weeks were injected once with AA under fed or 24-h fasted conditions, and the hypothalamus was collected 4 h after the injection: AA levels measured by LC–MS/MS in the hypothalamus (*n* = 3) (**a**); relative mRNA levels of hypothalamic neuropeptides (*n* = 6) (**b**). **c**, **d** Relative mRNA levels of (**c**) *Npy* and (**d**) *Agrp* upon 1 mM AA treatment up to 24 h (*n* = 3). **e**, **f** SVCT2 was pharmacologically inhibited by pretreatment of N41 cells with 1 mM sulfinpyrazone for 1 h under normal (Glu +) or glucose-deprived conditions (Glu –), followed by treatment with 1 mM AA for 4 h (*n* = 3): intracellular levels of ^13^C-labeled AA measured by LC–MS/MS (**e**) and relative mRNA levels of *Npy* (**f**). **g**, **h** SVCT2 was genetically inhibited by siRNA-mediated KD in N41 cells under normal (Glu +) or glucose-deprived conditions (Glu –), followed by treatment with 1 mM AA for 4 h (*n* = 3): intracellular levels of ^13^C-labeled AA measured by LC–MS/MS (**g**) and relative mRNA levels of *Npy* (**h**). Statistical significance was determined by two-way ANOVA followed by a post-hoc Tukey test. *P < 0.05, **P < 0.01, ***P < 0.001, ****P < 0.0001; nd not detected. Data are mean ± s.e.m.

The ARC of the hypothalamus serves as a central integrative center that regulates food intake through modulation of neuropeptide expression^41–44^. Given the robust suppression of food intake with weight loss under IF (Fig. 1b, c and Supplementary Fig. 3), we next investigated whether ARC hypothalamic mechanisms contribute to these effects. Dose-ranging studies of AA administration in DIO mice under AL feeding revealed a significant elevation in AA levels in the hypothalamus starting at 7.5 mmol/kg, with a greater increase observed at 15 mmol/kg (Supplementary Fig. 4a). A similar dose-dependent pattern was observed in food intake, with the strongest suppression occurring at 15 mmol/kg AA (Supplementary Fig. 4b). To further investigate a link between food intake and neuropeptide expression in AA treatment, we administered a single i.p. injection of Veh or AA to DIO mice under either fed or 24-h fasted conditions and subsequently assessed hypothalamic AA levels and neuropeptide expression. In fed mice, AA at 15 mmol/kg, but not 5 mmol/kg, significantly increased AA levels in the hypothalamus, with a greater elevation under fasting conditions (Fig. 2a). In the fed state, AA at 15 mmol/kg, but not 5 mmol/kg, downregulated the expression of the orexigenic neuropeptide genes *Npy* and *Agrp* in comparison with Veh (Fig. 2b). In the fasted state, AA more effectively downregulated *Npy* and *Agrp* expression, partially reversing their fasting-induced upregulation (Fig. 2b). By contrast, the expression of the anorexigenic neuropeptide genes *Pomc* and *Cart* was not significantly affected by AA treatment in either the fed or fasted group (Supplementary Fig. 4c). These findings suggest that an i.p. dose exceeding 5 mmol/kg of AA is required to elicit a notable increase in AA levels in the hypothalamus and downregulate *Npy* and *Agrp* expression. Moreover, fasting in combination with AA further elevates AA levels in the hypothalamus, leading to a more pronounced suppression of orexigenic neuropeptide expression.

To determine whether altered neuropeptide expression leads to changes in feeding behavior, we examined feeding patterns during the first 24 h following AA administration in DIO mice under AL feeding and measured *Npy*/*Agrp* expression at multiple time points. AA significantly suppressed food intake during the 3–6 h and 6–12 h periods, which coincided with downregulation of *Npy/Agrp* expression at 4 and 8 h, respectively (Supplementary Fig. 4d–f). These findings support a link between hypothalamic neuropeptide modulation and changes in feeding behavior.

To determine whether AA directly downregulates orexigenic neuropeptide expression, we treated N41, a hypothalamic cell line expressing *Npy* and *Agrp*, with 1 mM AA (a nontoxic concentration; Supplementary Fig. 5a) for up to 24 h and examined changes in *Npy* and *Agrp* expression. Consistent with the in vivo data, AA significantly reduced the mRNA levels of both *Npy* and *Agrp* (Fig. 2c, d). The most pronounced reduction in *Npy* expression was observed after 4 h treatment with AA; these conditions were subsequently used to confirm the anorexigenic effects of AA.

Because fasting induces a glucose-deprived state^56^, we examined the effect of fasting on AA-mediated downregulation of *Npy* expression by treating N41 cells with AA under glucose-deprived conditions (Glc –). These conditions, defined by 0.67 mM glucose, reflect residual glucose present in FBS and approximate hypothalamic glucose levels during fasting^57^. Therefore, our results represent physiological fasting responses rather than nonphysiological stress responses associated with complete glucose deprivation. To further elucidate the role of AA transporter in AA-mediated downregulation of *Npy* expression, we used pharmacological inhibition or gene KD of the sodium-dependent vitamin C transporter 2 (SVCT2), a neuronal AA transporter^58^. *Svct2* was predominantly expressed over *Svct1* in both N41 cells and hypothalamic tissue (Supplementary Fig. 6a), and the efficiency of *Svct2* KD was validated (Supplementary Fig. 6b).

Consistent with in vivo findings, intracellular AA levels (measured using [^13^C_6_]-AA) were higher under glucose-deprived conditions than under normal glucose conditions (Glc +) (Fig. 2e, g). Inhibition of AA transport with sulfinpyrazone, an SVCT2 inhibitor, or *Svct2* KD prevented the increase in intracellular AA levels, indicating that SVCT2 mediates AA uptake in N41 cells (Fig. 2e, g). Concurrently, the AA-induced *Npy* downregulation was abolished by inhibiting AA transport under both normal and glucose-deprived conditions (Fig. 2f, h). These results indicate that glucose deprivation increases AA uptake in N41 cells via SVCT2, thereby amplifying the downregulation of *Npy* expression.

To investigate the underlying mechanism of elevated AA uptake under fasting, we analyzed the expression of SVCT2 in both fasted DIO mice and glucose-deprived N41 cells. Fasting for 24 h significantly increased *Svct2* mRNA levels compared with fed controls (Supplementary Fig. 6c). Consistent with in vivo data, glucose deprivation for 4 h elevated both *Svct2* mRNA and SVCT2 protein levels in N41 cells (Supplementary Fig. 6d, e). These findings suggest that fasting-induced upregulation of SVCT2 drives enhanced AA uptake.

### AA is metabolized in the hypothalamus

**Fig. 3:**
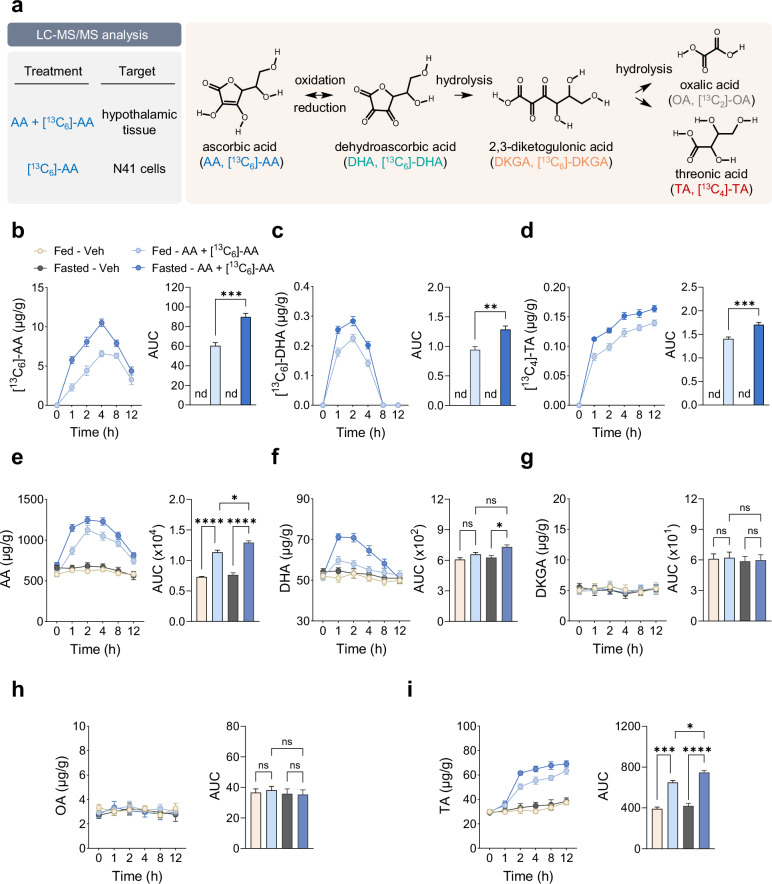
AA is metabolized in the hypothalamus of DIO mice. **a** Metabolic pathway of AA. **b**–**i** Metabolic flow of ^13^C-labeled and unlabeled AA in the hypothalamus of male DIO mice under fed or 24-h fasted conditions, as measured by LC–MS/MS following a single i.p. injection of a mixture of 13.5 mmol/kg AA and 1.5 mmol/kg [^13^C_6_]-AA (*n* = 3): ^13^C-labeled AA ([^13^C_6_]-AA) (**b**), ^13^C-labeled DHA ([^13^C_6_]-DHA) (**c**), ^13^C-labeled TA ([^13^C_4_]-TA) (**d**), AA (**e**), DHA (**f**), DKGA (**g**), OA (**h**) and TA (**i**). Statistical significance was determined by two-tailed unpaired Student’s *t*-test. *P < 0.05, **P < 0.01, ***P < 0.001, ****P < 0.0001; ns, no significance; nd, not detected; AUC, area under the curve. Data are mean ± s.e.m.

As outlined in Fig. 3a, AA undergoes sequential metabolism into DHA, DKGA, OA and TA^34^. However, the metabolic fate of AA upon entry into the hypothalamus remains unexplored. To trace the metabolic flux of AA in the hypothalamus, we administered a single i.p. injection of [^13^C_6_]-AA along with unlabeled AA to fed or 24-h fasted DIO mice. Targeted analysis of AA-derived metabolites using LC–MS/MS revealed that [^13^C_6_]-AA in the hypothalamus peaked at 4 h post-injection and gradually declined, accompanied by the detection of ^13^C-labeled metabolites [^13^C_6_]-DHA and [^13^C_4_]-TA in the fed group (Fig. 3b–d). The levels of [^13^C_6_]-AA and its ^13^C-labeled metabolites were higher in the fasted than in the fed group (Fig. 3b–d). ^13^C-labeled DKGA and OA were not detected, as their concentrations were below the quantitation limits of 5.3 μM and 1.6 μM, respectively (data not shown). Consistent with these data, analysis of the unlabeled AA metabolism also revealed higher levels of AA, DHA and TA in the hypothalamus of the fasted group compared with the fed group (Fig. 3e–i).

To determine the metabolic flow of AA in vitro, we treated N41 cells with 1 mM [^13^C_6_]-AA for up to 24 h and analyzed ^13^C-labeled metabolites. Intracellular levels of [^13^C_6_]-AA peaked at 4–6 h and subsequently declined, generating the ^13^C-labeled metabolites [^13^C_6_]-DHA, [^13^C_6_]-DKGA and [^13^C_4_]-TA (Supplementary Fig. 7a–e). The [^13^C_6_]-DKGA levels (Supplementary Fig. 7c) were near the quantitation limit and [^13^C_2_]-OA was undetectable, similar to in vivo observations.

To examine the effect of fasting on AA metabolism, we treated N41 cells with 1 mM [^13^C_6_]-AA under normal or glucose-deprived conditions. The intracellular levels of [^13^C_6_]-AA, [^13^C_6_]-DHA, [^13^C_6_]-DKGA and [^13^C_4_]-TA were significantly higher after glucose deprivation for 4 h than under normal glucose conditions (Supplementary Fig. 7f–i). Collectively, these results demonstrate that, once transported to the hypothalamus, AA is metabolized into DHA, DKGA and TA, and their levels are elevated under fasting conditions.

### DHA and TA downregulate hypothalamic *Npy/Agrp* expression

AA metabolites offer distinct metabolic benefits beyond those of AA^35,36^, raising the question of whether individual AA metabolites contribute to the regulation of *Npy*/*Agrp* expression^36,59^. To address this question, we treated N41 cells with nontoxic concentrations (1 mM) of DHA, DKGA or TA (Supplementary Fig. 5b) and examined their effects on AA metabolism and *Npy*/*Agrp* expression.

**Fig. 4:**
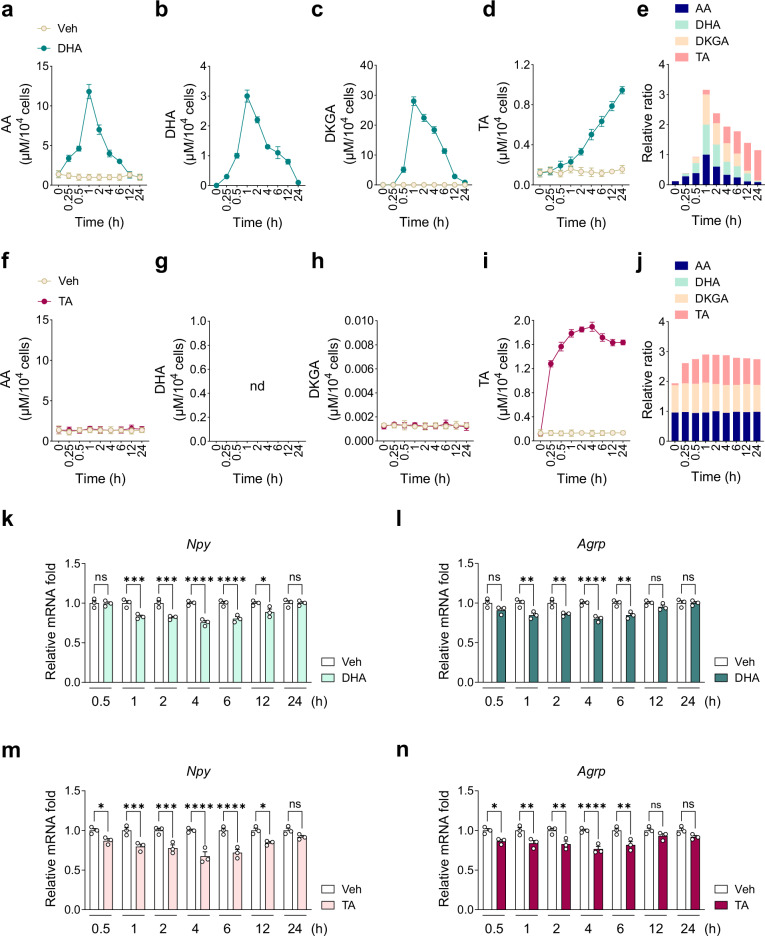
DHA and TA downregulate *Npy* and *Agrp* expression in N41 cells. **a**–**e** Metabolic flow of 1 mM DHA (*n* = 3): AA (**a**), DHA (**b**), DKGA (**c**), TA (**d**) and relative ratios of AA and its metabolites (**e**). **f**–**j** Metabolic flow of 1 mM TA (*n* = 3): AA (**f**), DHA (**g**), DKGA (**h**), TA (**i**) and relative ratios of AA and its metabolites (**j**). **k**, **l** Relative mRNA levels of *Npy* (**k**) and *Agrp* (**l**) upon treatment with 1 mM DHA for up to 24 h (*n* = 3). **m**, **n** Relative mRNA levels of *Npy* (**m**) and *Agrp* (**n**) upon treatment with 1 mM TA for up to 24 h (*n* = 3). Statistical significance was determined by two-way ANOVA followed by a post-hoc Tukey test. *P < 0.05, ***P < 0.001, ****P < 0.0001; ns, no significance; nd, not detected. Data are mean ± s.e.m.

Following DHA treatment, intracellular levels of AA, DHA and DKGA peaked at 1 h and gradually declined, whereas TA levels showed a sustained increase throughout the observation period (Fig. 4a–e). Concurrently, DHA treatment significantly downregulated *Npy* expression between 1 h and 12 h, and *Agrp* expression between 1 h and 6 h (Fig. 4k, l). Of note, DHA treatment increased intracellular AA levels within 1 h (Fig. 4a), as DHA can be converted back to AA through intracellular reduction^34^. In DHA treatment, intracellular DHA levels peaked earlier (1 h; Fig. 4b) than did intracellular AA levels in AA treatment (4–6 h; Supplementary Fig. 7a), consistent with previous findings that DHA uptake via glucose transporters (GLUTs) is faster than AA uptake via SVCTs due to the passive gradient-driven nature of GLUTs, unlike the active energy-dependent mechanism of SVCTs^60,61^. This rapid uptake, followed by subsequent metabolism into TA, may account for the earlier downregulation of *Npy* observed at 1 h with DHA (Fig. 4k), compared with 2 h with AA (Fig. 2c). DKGA treatment had no effect on intracellular AA metabolite levels or *Npy* expression (Supplementary Fig. 8).

TA treatment increased intracellular TA levels, which peaked at 4 h, without changes in other AA metabolites (Fig. 4f–j). Notably, TA treatment downregulated *Npy* expression much earlier (starting at 0.5 h) and at lower concentrations (0.01 and 0.1 mM) than did AA (Fig. 4m and Supplementary Fig. 9). TA treatment also significantly downregulated *Agrp* expression between 0.5 h and 6 h (Fig. 4n). These findings indicate that DHA and TA downregulate *Npy*/*Agrp* expression more effectively than does AA.

### TA is a key metabolite downregulating *Npy*/*Agrp* expression

Modulating AA metabolism is challenging because most steps are nonenzymatic, except for the reversible interconversion between AA and DHA, which is governed by oxidation and reduction^34^. Intracellular reducing agents such as GSH-MEE can convert DHA back to AA, thereby blocking the metabolism of DHA into DKGA, OA and TA^34^. To identify the key metabolite responsible for downregulating *Npy*/*Agrp* expression, we treated N41 cells with AA, DHA or TA in the presence or absence of GSH-MEE and analyzed changes in *Npy*/*Agrp* expression and AA metabolite levels.

**Fig. 5:**
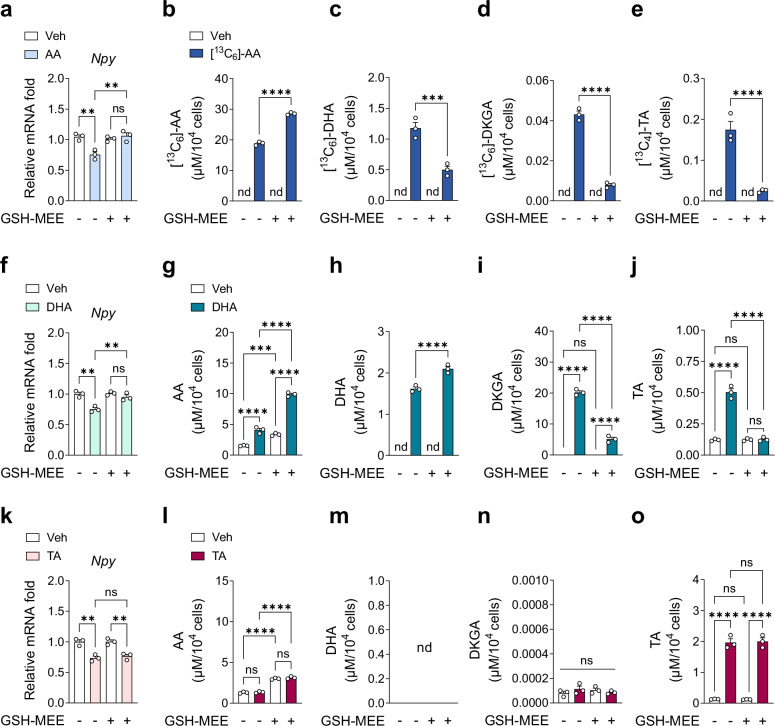
TA is a key metabolite responsible for the downregulation of *Npy* expression in N41 cells. **a**–**e** Relative mRNA levels of *Npy* (**a**) and metabolic flow of AA after treatment with 1 mM AA for 4 h (**b**–**e**), in the presence or absence of intracellular reducing agent (1 mM GSH-MEE, pretreatment for 12 h) (*n* = 3): [^13^C_6_]-AA (**b**), [^13^C_6_]-DHA (**c**), [^13^C_6_]-DKGA (**d**) and [^13^C_4_]-TA (**e**). **f**–**j** Relative mRNA levels of *Npy* (**f**) and metabolic flow of DHA after treatment with 1 mM DHA for 4 h (**g**–**j**), in the presence or absence of GSH-MEE (*n* = 3): AA (**g**), DHA (**h**), DKGA (**i**) and TA (**j**). **k**–**o** Relative mRNA levels of *Npy* (**k**) and metabolic flow of TA after treatment with 1 mM TA for 4 h (**l**–**o**), in the presence or absence of GSH-MEE (*n* = 3): AA (**l**), DHA (**m**), DKGA (**n**) and TA (**o**). Statistical significance was determined by two-tailed unpaired Student’s *t*-test in **b**–**e** and two-way ANOVA followed by a post-hoc Tukey test in **g**–**j** and **l**–**o**. **P < 0.01, ***P < 0.001, ****P < 0.0001; ns, no significance; nd, not detected. Data are mean ± s.e.m.

GSH-MEE abolished the AA-induced downregulation of *Npy* and *Agrp* expression and induced accumulation of intracellular AA and a decrease in the levels of its metabolites (Fig. 5a–e and Supplementary Fig. 10a). Similarly, GSH-MEE abolished the DHA-induced downregulation of *Npy* and *Agrp* expression, while increasing intracellular levels of both AA and DHA but reducing those of downstream metabolites (Fig. 5f–j and Supplementary Fig. 10b). In the presence of GSH-MEE, intracellular DHA levels remained elevated following DHA treatment, probably due to the high levels of reduced AA converting back to DHA to maintain equilibrium (Fig. 5h). These results suggest that elevated intracellular levels of AA or DHA are not responsible for the suppression of *Npy*/*Agrp* expression. By contrast, TA downregulated *Npy* and *Agrp* expression in the presence of GSH-MEE, without altering the levels of other AA metabolites (Fig. 5k–o and Supplementary Fig. 10c). Together, these findings suggest that TA, rather than AA or DHA, is the key mediator of *Npy*/*Agrp* downregulation.

Given that AA and its metabolites may have antioxidant or pro-oxidant properties^62,63^, we investigated whether the observed changes in *Npy*/*Agrp* expression were associated with alterations in cellular redox status. We measured ROS levels in N41 cells treated with AA, DHA or TA for 4 h in the presence or absence of GSH-MEE. Consistent with the known pro-oxidant effects of DHA^62^, DHA treatment increased intracellular ROS levels, and this effect was abolished by GSH-MEE (Supplementary Fig. 11a, c, e). Neither AA nor TA significantly affected ROS levels under our experimental conditions (Supplementary Fig. 11b, d, e). These results suggest that the downregulation of *Npy*/*Agrp* expression by AA or TA was not mediated by changes in the cellular redox status.

### TA and IF synergistically ameliorate obesity in DIO mice

**Fig. 6:**
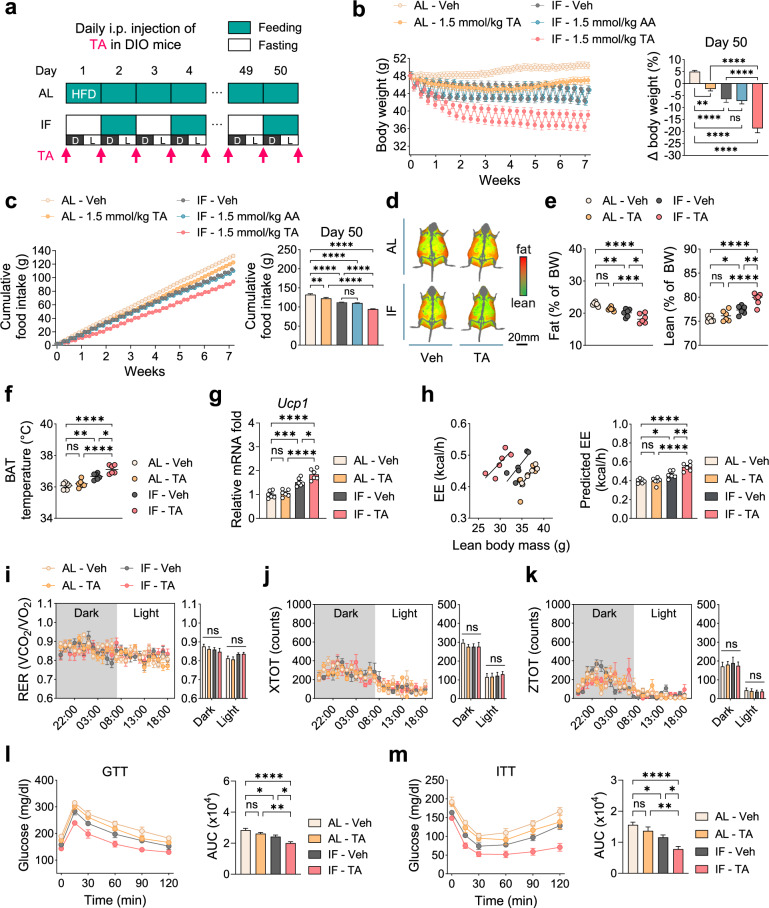
TA in combination with IF exerts a synergistic anti-obesity effect in DIO mice. **a** Design of i.p. injections. **b**, **c** Changes in body weight measured daily (left) and on day 50 (relative to day 0, right) (**b**) and cumulative food intake measured daily (left) and on day 50 (relative to day 0, right) (**c**) in male mice injected intraperitoneally with TA (under fed or 24-h fasted conditions) or AA (under 24-h fasted conditions only) (*n* = 6). **d** Representative images of body composition. Red, fat mass; green, lean mass. **e** Fat mass (left) and lean mass (right) on day 50 (*n* = 6). **f** Temperature of interscapular BAT (*n* = 6). **g** Relative mRNA levels of *Ucp1* in interscapular BAT (*n* = 6). **h** Left: regression-based analysis of EE against lean body mass. Right: bar graph indicating EE values adjusted for lean body mass using ANCOVA (*n* = 6). **i**–**k** Indirect calorimetry parameters: RER (**i**), locomotor activity (*X* axis) (**j**) and locomotor activity (*Z* axis) (**k**) (*n* = 6). **l** Glucose tolerance test (GTT) (*n* = 6). **m**, Insulin tolerance test (ITT) (*n* = 6). Statistical significance was determined by two-way ANOVA followed by a post-hoc Tukey test. *P < 0.05, **P < 0.01, ***P < 0.001, ****P < 0.0001; ns, no significance; AUC, area under the curve; D, dark phase; L, light phase. Data are mean ± s.e.m.

To evaluate the therapeutic efficacy of TA in mitigating obesity, DIO mice fed an HFD for 16 weeks were subjected to either AL or IF regimen for 50 days, with daily i.p. injections of either Veh or TA (Fig. 6a). We compared the metabolic parameters across Veh- and TA-administered groups under AL and IF conditions: AL-Veh, AL-TA (1.5 mmol/kg), IF-Veh and IF-TA (1.5 mmol/kg). Due to the poor solubility of TA, a dose of 1.5 mmol/kg (10-fold lower than the AA dose used) was administered, with no signs of sickness observed over the 50-day injection period.

In male DIO mice, administration of TA under AL feeding led to a modest reduction in food intake on day 1 compared with Veh controls (Supplementary Fig. 12a). Sustained modest reductions in daily food intake over 50 days resulted in significant decreases in cumulative food intake and body weight in TA-treated mice under AL feeding compared with Veh controls (Fig. 6b, c). On day 2, the combination of TA and IF produced a greater reduction in food intake and body weight compared with TA or IF alone (Supplementary Fig. 12). On day 50, IF combined with TA significantly reduced body weight (23.7%) and food intake (28.9%) in comparison with AL regimen, surpassing the effects of TA or IF alone (Fig. 6b, c). To directly compare the therapeutic efficacy of TA and AA in mitigating obesity, we included an additional group (1.5 mmol/kg AA under IF). Notably, this dose of AA did not affect body weight or food intake (Fig. 6b, c), suggesting that the combination of IF with TA is more effective than that with AA in ameliorating obesity.

We also observed that DIO mice undergoing IF combined with TA had significantly lower fat mass and elevated interscapular surface temperature and *Ucp1* mRNA levels in BAT compared with mice treated with IF or TA alone (Fig. 6d–g). Indirect calorimetry revealed increased EE in the IF with TA treatment compared with IF or TA alone, with no changes in RER or locomotor activity (Fig. 6h–k). Furthermore, IF combined with TA significantly improved glucose tolerance and insulin sensitivity compared with either IF or TA alone (Fig. 6l, m). Collectively, these findings demonstrate that IF combined with TA has more pronounced anti-obesity effects than either intervention alone in DIO mice.

In female DIO mice, the combination of IF and TA induced metabolic changes similar to those observed in males, including greater reduction in body weight (21.5%) and food intake (26.1%), as well as increased EE compared with either intervention alone (Supplementary Fig. 13).

### TA uptake via GLUTs is elevated under fasting, resulting in downregulation of orexigenic neuropeptide expression in the hypothalamus

**Fig. 7:**
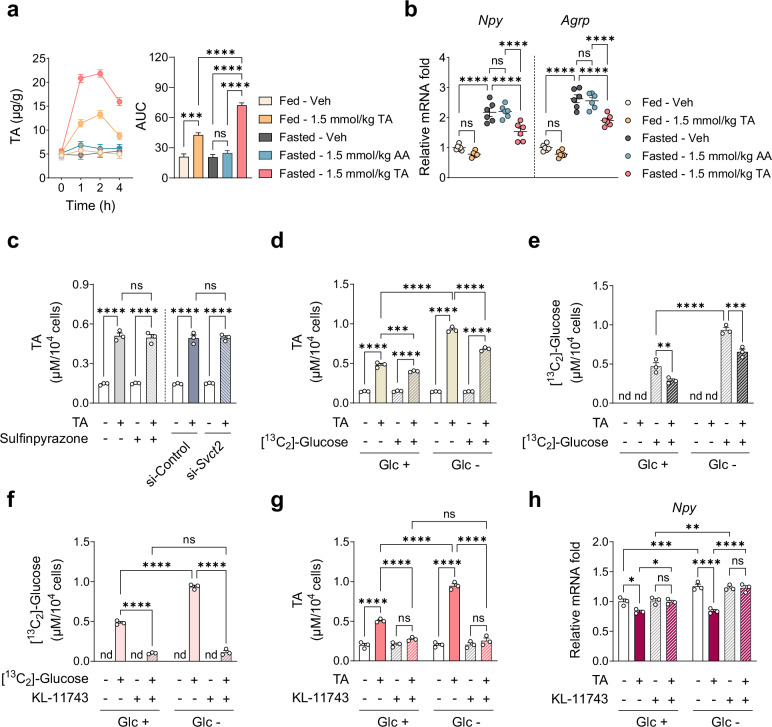
Elevated TA uptake via GLUTs during fasting downregulates expression of orexigenic neuropeptides in the hypothalamus and N41 cells. **a** TA levels in the hypothalamus (*n* = 3). **b** Relative mRNA levels of hypothalamic neuropeptides. RNA was isolated from the hypothalamus of male DIO mice collected 4 h after a single i.p. injection of TA (under fed or 24-h fasted conditions) or AA (under 24-h fasted conditions only) (*n* = 6). **c** Relative intracellular TA levels upon 0.1 mM TA treatment for 1 h; SVCT2 was pharmacologically inhibited by 1 mM sulfinpyrazone for 1 h or was genetically inhibited by siRNA-mediated KD in N41 cells (*n* = 3). **d**, **e** N41 cells were cotreated with 0.1 mM TA and 0.1 mM ^13^C-labeled glucose under normal (Glu +) or glucose-deprived conditions (Glu –) (*n* = 3): TA levels (**d**) and ^13^C-labeled glucose levels (**e**). **f**–**h** N41 cells were treated with 0.1 mM TA for 1 h in the presence or absence of a GLUT inhibitor (0.1 mM KL-11743, pretreatment for 1 h) under normal (Glu +) or glucose-deprived conditions (Glu –) (*n* = 3): ^13^C-labeled glucose levels (**f**), TA levels (**g**) and relative mRNA levels of *Npy* (**h**). Statistical significance was determined by two-way ANOVA followed by a post-hoc Tukey test. *P < 0.05, **P < 0.01, ***P < 0.001, ****P < 0.0001; ns, no significance; nd, not detected; AUC, area under the curve. Data are mean ± s.e.m.

To elucidate the anorexigenic effects of TA, DIO mice were administered a single i.p. injection of Veh or TA under fed or 24-h fasted conditions, and changes in AA metabolite levels and neuropeptide expression in the hypothalamus were evaluated simultaneously. Administration of 1.5 mmol/kg TA significantly increased TA levels in the hypothalamus within 4 h post-injection, and fasting further elevated these levels (Fig. 7a). Administration of TA did not alter the levels of other AA metabolites (Supplementary Fig. 14). Under fed conditions, 1.5 mmol/kg TA induced a slight but nonsignificant reduction in *Npy* and *Agrp* expression compared with the control (Fig. 7b). By contrast, TA significantly downregulated *Npy* and *Agrp* expression under fasting conditions, effectively reversing their fasting-induced upregulation (Fig. 7b). Conversely, TA had no effect on the mRNA levels of *Pomc* and *Cart* in either the fed or fasted group (Supplementary Fig. 15a). We further analyzed feeding patterns during the first 24 h following TA administration in DIO mice under AL feeding, and measured *Npy*/*Agrp* expression at multiple time points. TA significantly suppressed food intake during the 3–6 h period, which was accompanied by reduced *Npy*/*Agrp* expression at 4 h (Supplementary Fig. 15b–d), supporting a connection between neuropeptide expression and feeding behavior. An equivalent dose of AA did not affect AA metabolite levels or the expression of orexigenic neuropeptides under fasted conditions (Fig. 7a, b and Supplementary Fig. 14). These results demonstrate that TA plays a critical role in modulating orexigenic neuropeptide expression in the hypothalamus.

To elucidate how TA levels are elevated in the hypothalamus during fasting, we first examined whether TA uptake is mediated by the same transporter as AA uptake in N41 cells. Inhibition of SVCT2 had no effect on TA uptake following TA treatment, indicating that SVCT2 does not facilitate TA transport (Fig. 7c). We next investigated whether TA uptake is influenced by glucose availability. We cotreated N41 cells with equimolar concentrations (0.1 mM) of TA and [^13^C_2_]-glucose under normal or glucose-deprived conditions and quantified the intracellular levels of TA and [^13^C_2_]-glucose. As the previously used dose of 1 mM TA led to a rapid saturation of intracellular TA within 1 h (Supplementary Fig. 9a), the TA concentration was reduced to 0.1 mM and intracellular uptake was measured at 1 h. TA treatment led to greater TA uptake under glucose deprivation than under normal glucose conditions, and cotreatment with [^13^C_2_]-glucose reduced TA uptake under both conditions (Fig. 7d). Similarly, [^13^C_2_]-glucose uptake was greater under glucose deprivation than under normal glucose conditions, whereas cotreatment with TA reduced its uptake under both conditions (Fig. 7e). These findings indicate that TA and glucose compete for cellular uptake, resulting in enhanced TA uptake when glucose is limited.

We next examined whether GLUTs facilitate TA uptake and mediate TA-induced *Npy*/*Agrp* downregulation during fasting. We treated N41 cells with TA under normal or glucose-deprived conditions in the presence or absence of KL-11743, a GLUT1-4 inhibitor, and analyzed changes in intracellular TA levels and *Npy*/*Agrp* expression. Pretreatment with KL-11743 effectively blocked glucose uptake under both normal and glucose-deprived conditions (Fig. 7f). TA treatment led to a greater increase in intracellular TA levels under glucose deprivation than under normal glucose conditions, but this effect was abolished by KL-11743 (Fig. 7g). Moreover, KL-11743 prevented TA-induced downregulation of *Npy* and *Agrp* expression under both conditions (Fig. 7h and Supplementary Fig. 10d). These findings indicate that, in comparison with fed conditions, the increased TA uptake through GLUTs during fasting promotes greater *Npy*/*Agrp* downregulation in the hypothalamus.

### GLUT3 is required for the enhancement of the anorexigenic effects of TA by fasting

During fasting, GLUT3 is upregulated in hypothalamic neurons, probably as a protective mechanism against energy depletion in the brain^64,65^. To determine the changes in GLUT3 levels induced by TA in hypothalamic NPY neurons under fasting conditions, we administered a single i.p. injection of TA to NPY-hrGFP DIO mice under fed or 24-h fasted conditions and measured the fluorescence intensities of GLUT3 and NPY in hrGFP-positive (hrGFP^+^) cells. Changes in the hrGFP signal reflect alterations in the transcriptional activity of NPY in *Npy*-expressing neurons, rather than quantitative changes in NPY expression itself.

**Fig. 8:**
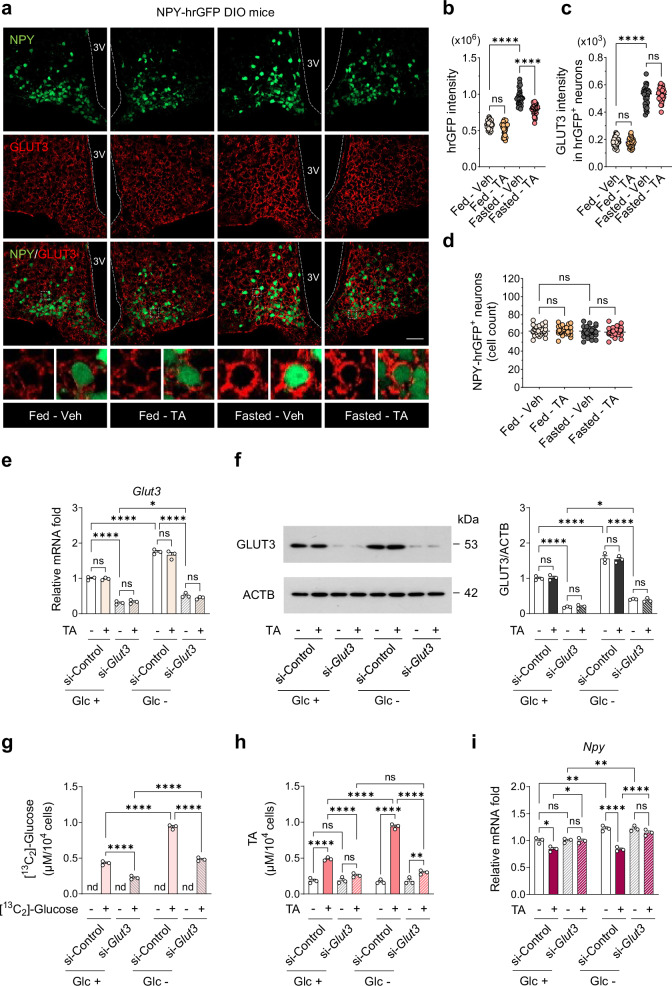
GLUT3 mediates the synergistic effects of TA under fasting. **a** Immunohistochemistry analysis of NPY and GLUT3 fluorescence intensity in the ARC of NPY-hrGFP DIO mice. Mice fed an HFD for 16 weeks received a single i.p. injection of 1.5 mmol/kg TA under fed or 24-h fasted conditions, and brains were harvested 4 h later. **b**–**d** Eight brain slices (35 μm thickness) were obtained from each of four mice per group (*n* = 32) to determine the intensity of hrGFP (**b**) and GLUT3 (**c**) in hrGFP-positive cells, and the number of hrGFP-positive cells (**d**). Scale bars, 20 μm. **e**–**i** GLUT3 was genetically inhibited by siRNA-mediated KD in N41 cells, and cells were treated with 0.1 mM TA for 1 h under normal (Glu +) or glucose-deprived conditions (Glu –) (*n* = 3): relative mRNA levels of *Glut3* (**e**), western blot analysis of GLUT3 (**f**), ^13^C-labeled glucose levels (**g**), TA levels (**h**) and relative mRNA levels of *Npy* (**i**). Statistical significance was determined by two-way ANOVA followed by a post-hoc Tukey test. *P < 0.05, **P < 0.01, ***P < 0.001, ****P < 0.0001; ns, no significance; nd, not detected. Data are mean ± s.e.m.

In the fed state, the fluorescence intensity of hrGFP remained unchanged in NPY-hrGFP DIO mice treated with TA (Fig. 8a, b). Fasting increased hrGFP fluorescence intensity, suggesting enhanced *Npy* transcriptional activity, but this effect was significantly reduced by TA (Fig. 8a, b). Fasting also increased GLUT3 fluorescence intensity in hrGFP^+^ cells, regardless of TA administration (Fig. 8a, c). The number of NPY-hrGFP^+^ cells was similar across all experimental groups (Fig. 8d). We also confirmed that a single i.p. administration of AA under fasted conditions led to a more pronounced reduction in hrGFP fluorescence intensity in NPY-hrGFP DIO mice compared with the fed state (Supplementary Fig. 16). Collectively, these results indicate that fasting induces GLUT3 expression in NPY neurons and that TA does not alter GLUT3 expression.

To corroborate these findings, we treated N41 cells with TA following *Glut3* KD under normal or glucose-deprived conditions. Consistent with the in vivo findings, glucose deprivation significantly increased both GLUT3 mRNA and protein levels, and this effect was not influenced by TA treatment (Fig. 8e, f). *Glut3* KD significantly attenuated the glucose-deprivation-induced upregulation of GLUT3 at both the mRNA and protein levels (Fig. 8e, f) and reduced glucose uptake under both normal and glucose-deprived conditions (Fig. 8g). Of note, *Glut3* KD markedly reduced TA uptake and abolished TA-mediated downregulation of *Npy* and *Agrp* expression under both conditions (Fig. 8h, i and Supplementary Fig. 10e). Overall, these findings indicate that GLUT3 is required for the enhanced anorexigenic effects of TA during fasting.

## Discussion

Both IF and AA face limitations as monotherapies for obesity treatment. For example, IF is often associated with exacerbated hypoglycemia when paired with glucose-lowering drugs such as insulin or glucagon-like peptide-1 receptor agonists^66–68^ and may lead to increased food cravings and nutrient deficiencies, particularly AA, B vitamins, iron and calcium^69,70^. Although AA shows metabolic benefits, clinical trials have not consistently demonstrated substantial weight loss^71,72^. This study investigated the combination of IF and AA for enhanced anti-obesity effects, explored their synergistic benefits and suggested TA as a more effective alternative owing to its potential to regulate systemic energy homeostasis via hypothalamic mechanisms.

The doses used in this study are pharmacological and do not represent typical physiological intake. Previous studies administering AA at physiological levels reported minimal efficacy in reducing obesity^72,73^. Given AA’s rapid metabolism and clearance, we hypothesized that sustained exposure at pharmacological levels might be necessary to achieve therapeutic effects in the context of diet-induced obesity. Indeed, our results demonstrated that the *Npy*/*Agrp* expression returned to baseline within 24 h following AA administration. This transient effect, consistently observed in both in vitro and in vivo studies, underscores the necessity of repeated dosing to maintain systemic exposure and achieve meaningful metabolic benefits. Notably, combining IF with AA or TA improved metabolic parameters in DIO mice including food intake, EE, body composition, glucose tolerance and insulin sensitivity, resulting in a greater weight loss compared with either intervention alone. Despite a 10-fold lower dose of TA than AA, TA combined with IF resulted in a 3.3-fold reduction in body weight compared with TA alone, whereas AA combined with IF led to a 2.3-fold reduction compared with AA alone. Furthermore, comparison at equimolar concentrations confirmed that TA was more effective than AA in mitigating obesity when combined with IF. Pair-feeding experiments would offer valuable insight into the contribution of reduced food intake to the overall phenotype. Future studies using pair-feeding designs will be essential to determine the extent to which AA and TA exert metabolic effects independent of caloric intake. TA’s superior efficacy over AA was also reflected in its profound downregulation of orexigenic neuropeptide expression, with a lower dose required and effects being achieved within a shorter timeframe. Intriguingly, a recent metabolomic profiling of serum from DIO mice revealed significantly lower TA levels than those in normal-chow-fed mice^74^. In the present study, we observed that TA administration elevated plasma TA levels in DIO mice, which were further increased when TA was combined with IF (data not shown). In parallel, clinical studies have reported that plasma AA levels are lower in individuals with obesity than in lean individuals, and that serum AA levels are inversely correlated with body mass index and waist circumference^73,75,76^. These observations suggest that restoring reduced AA levels may contribute to the prevention and treatment of obesity. In this context, our findings suggest that TA supplementation may mitigate obesity, potentially through its hypothalamic regulation of energy homeostasis.

Here, we propose that AA and its metabolites may play a distinct role in the regulation of energy homeostasis, potentially acting within the hypothalamus. However, our study does not provide direct evidence of hypothalamus-specific enrichment of AA or TA, and extrahypothalamic mechanisms may also contribute to the observed effects. To clarify the site-specific contributions, future studies using direct brain infusion via intracerebroventricular or cerebrospinal fluid infusion with isotope-labeled AA and TA tracing will be essential. In addition, expanding analyses to brain regions such as the paraventricular nucleus, lateral hypothalamus or nucleus accumbens may provide complementary insights into alternative pathways involved in the central regulation of energy homeostasis. In our study, 15 mmol/kg AA produced greater weight loss and a significant increase in hypothalamic AA levels compared with a 5 mmol/kg dose, which did not elevate hypothalamic AA despite modest weight reduction. These findings suggest that surpassing a threshold concentration of AA in the hypothalamus may be necessary to engage central mechanisms that enhance its therapeutic potential, and dosing strategies should aim to achieve effective hypothalamic engagement while remaining feasible and safe for clinical application.

Using a targeted metabolomics approach, we traced the metabolic fate of AA and found a sustained increase in TA levels but transient increases in the other AA metabolites in the hypothalamic tissue and N41 cells. Treatment with AA, DHA or TA downregulated *Npy*/*Agrp* expression in N41 cells, whereas GSH-MEE, which reduces DHA back to AA, abolished the *Npy*/*Agrp* downregulation by AA and DHA. This suggests that *Npy*/*Agrp* expression is not directly suppressed by AA or DHA, but rather depends on the formation of downstream metabolites. By contrast, TA downregulated *Npy*/*Agrp* expression even when AA metabolism was blocked, indicating that TA is the key AA metabolite that downregulates *Npy*/*Agrp*. In our study, TA administration under fasting conditions led to a more pronounced downregulation of *Npy*/*Agrp* expression, while *Pomc*/*Cart* expression remained unaffected. Administration of TA during fasting may mitigate excessive hunger and food cravings, which are frequently reported adverse effects of IF^70,77^. By attenuating *Npy*/*Agrp* expression during this phase, AA and TA treatment may modulate the anticipatory drive to eat, thereby influencing subsequent feeding behavior. To our knowledge, the mechanisms by which TA regulates gene expression in neurons remain largely unexplored. However, in the ARC of the hypothalamus, fasting has been shown to stimulate CRE-mediated gene induction, leading to increased *Npy* mRNA levels via enhanced phosphorylation of CRE-binding protein^78^. Notably, this fasting-induced CRE activation occurs specifically in NPY neurons, but not in POMC neurons. In addition, fasting upregulates *Npy* expression through AMPK activation, which promotes autophagy or calcium influx in NPY/AGRP neurons^79,80^. We speculate that TA may counteract one or more of these fasting-associated signaling mechanisms in NPY/AGRP neurons, thereby selectively regulating *Npy*/*Agrp* gene expression. Further research is needed to test this possibility and to elucidate the downstream effects of TA-fasting synergy on hypothalamic regulation of energy balance.

Our findings demonstrate that TA competes with glucose for uptake via GLUT3 in the hypothalamus. This is in line with a previous report that pharmacological inhibition of GLUTs abolishes TA-induced increases in synaptic density and intracellular magnesium levels in hippocampal neurons^81^. We also observed fasting-induced upregulation of GLUT3 expression in NPY neurons in NPY-hrGFP DIO mice. Given that global or neural-specific GLUT3 knockout models result in embryonic lethality or neurobehavioral abnormalities^82–84^, we used a GLUT3 KD approach in N41 cells to investigate its functional role. This strategy attenuated glucose uptake while preserving cellular responsiveness to glucose availability such as the fasting-induced increase in *Npy*/*Agrp* expression. We found that TA uptake in N41 cells was enhanced under glucose-deprived conditions, due to both the absence of glucose and glucose deprivation-induced upregulation of GLUT3. The resulting increase in intracellular TA led to a more pronounced downregulation of *Npy*/*Agrp* expression, which may account for the synergistic improvement in whole-body energy homeostasis observed when IF was combined with TA. We also provide evidence for a potent synergistic mechanism by which IF enhances the effects of AA through upregulation of SVCT2 in the hypothalamus. In colorectal cancer cells, AA treatment under low-glucose conditions exerted a synergistic anticancer effect by enhancing DHA uptake via GLUT1, leading to elevated intracellular oxidative stress and subsequent apoptosis^62^. By contrast, our findings indicate that AA uptake in N41 cells is not mediated by GLUT1 or GLUT3 (data not shown), but occurs through SVCT2.

In conclusion, our study demonstrates that TA, an AA metabolite, exerts anti-obesity effects, at least in part by regulating hypothalamic neuropeptides, and acts synergistically when combined with IF. Identification of the differences in the regulation of TA and AA transport by glucose deprivation in the hypothalamus (via GLUT3 and SVCT2, respectively) provides insight into the mechanisms underlying this synergism. The alternate-day fasting IF model used in this study was designed to maximize hunger, allowing appetite-reducing interventions to demonstrate their full efficacy. Future studies may need to refine TA administration strategies, including evaluation of less-intensive dosing schedules and potential rebound effects following treatment withdrawal in various IF models, to further harness its anti-obesity effects. Our study advances the understanding of AA metabolism in the hypothalamus and highlights the potential of combining IF with TA as a novel and sustainable strategy for obesity treatment.

## Supplementary information

Supplementary Information
