# Supplementary material for: Threonic acid, an ascorbic acid metabolite, synergizes with intermittent fasting to ameliorate obesity
Source: Exp Mol Med. 2026 Jan 9;58(1):126–42. doi: 10.1038/s12276-025-01613-y (PMC12868866; doi:10.1038/s12276-025-01613-y)
Supplement: Supplementary file 1 — Supplementary Information [file 12276_2025_1613_MOESM1_ESM.pdf]

Supplementary Information

Threonic acid, an ascorbic acid metabolite, synergizes with intermittent fasting to ameliorate obesity

Sungjoon Oh, Seokjae Park and Eun-Kyoung Kim

Supplementary Figure 1

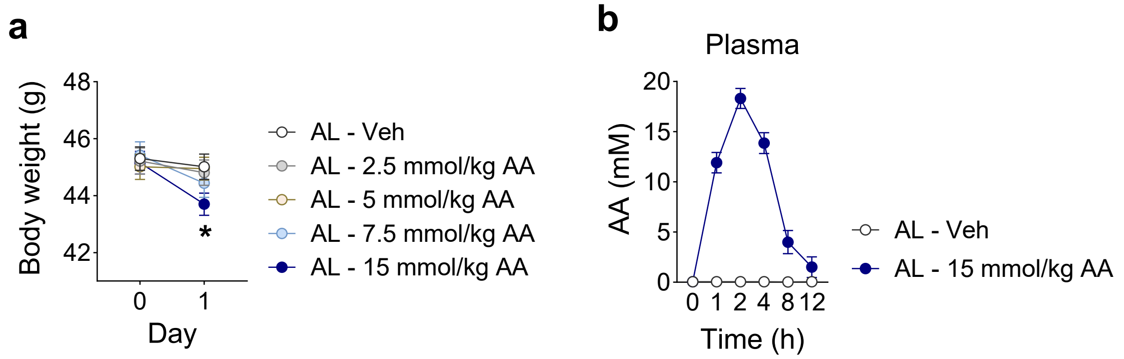

Supplementary Figure 1. Effects of a single administration of AA on body weight and plasma AA levels in male DIO mice. **a** DIO mice received a single i.p. administration of 2.5, 5, 7.5, or 15 mmol/kg AA under AL feeding, and changes in body weight were measured daily (n=3). **b** Changes in plasma AA levels measured by LC-MS/MS following a single i.p. administration of 15 mmol/kg AA in DIO mice (n=3). Statistical significance was determined by one-way ANOVA followed by a *post hoc* Tukey test. *$P < 0.05$; vehicle vs 15 mmol/kg AA. Data are mean ± SEM.

Supplementary Figure 2

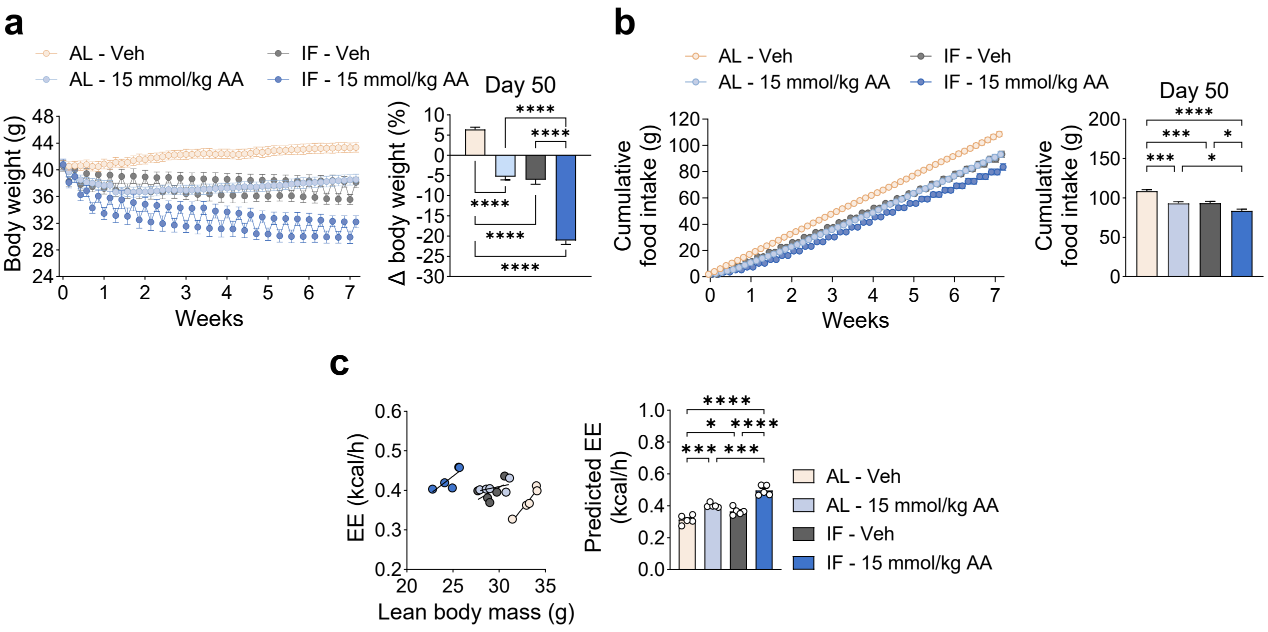

Supplementary Figure 2. Administration of AA in combination with IF enhances anti-obesity effects in female DIO mice. a–c DIO mice were subjected to either AL or IF regimen for 50 days, with daily i.p. injections of Veh or 15 mmol/kg AA (n=5). Changes in **(a)** body weight measured daily (left panel) and on day 50 (relative to day 0, right panel), **(b)** cumulative food intake measured daily (left panel) and on day 50 (relative to day 0, right panel), and **(c)** regression-based analysis of energy expenditure (EE) against lean body mass (left panel) and EE values adjusted for lean body mass using ANCOVA (right panel). Statistical significance was determined by two-way ANOVA followed by a *post hoc* Tukey test. $*P < 0.05$, $***P < 0.001$, $****P < 0.0001$; ns, no significance. Data are mean ± SEM.

Supplementary Figure 3

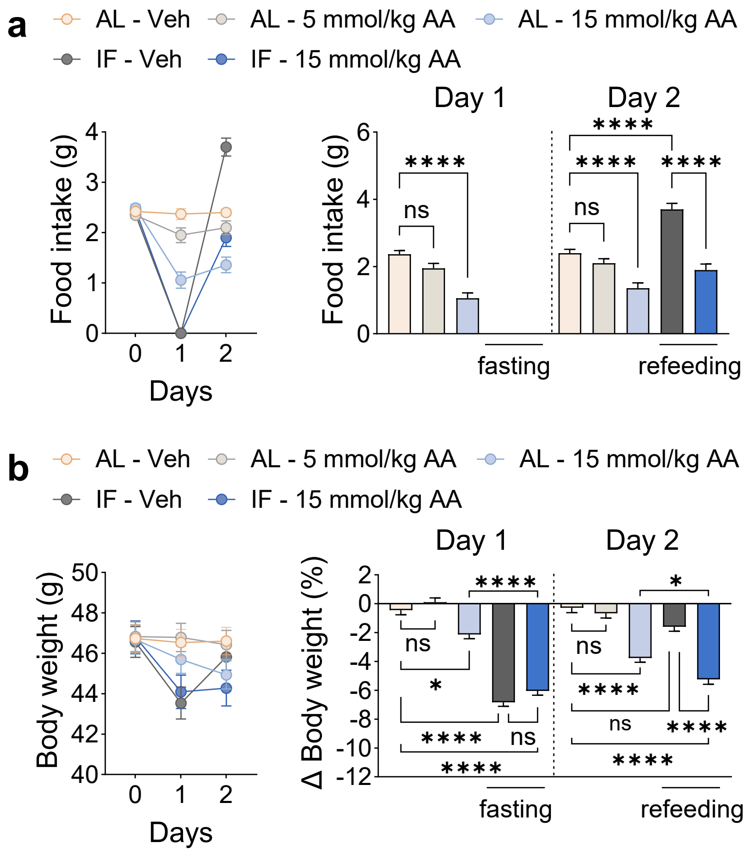

Supplementary Figure 3. Acute effects of AA administration on food intake and body weight under either AL or IF in male DIO mice. a, b DIO mice were subjected to either AL or IF regimen for 50 days, with daily i.p. injections of Veh or AA (5 or 15 mmol/kg). On days 0, 1, and 2, changes in **(a)** daily food intake and **(b)** body weight (n=6). Statistical significance was determined by two-way ANOVA followed by a *post hoc* Tukey test. *P < 0.05, ****P < 0.0001; ns, no significance. Data are mean ± SEM.

Supplementary Figure 4

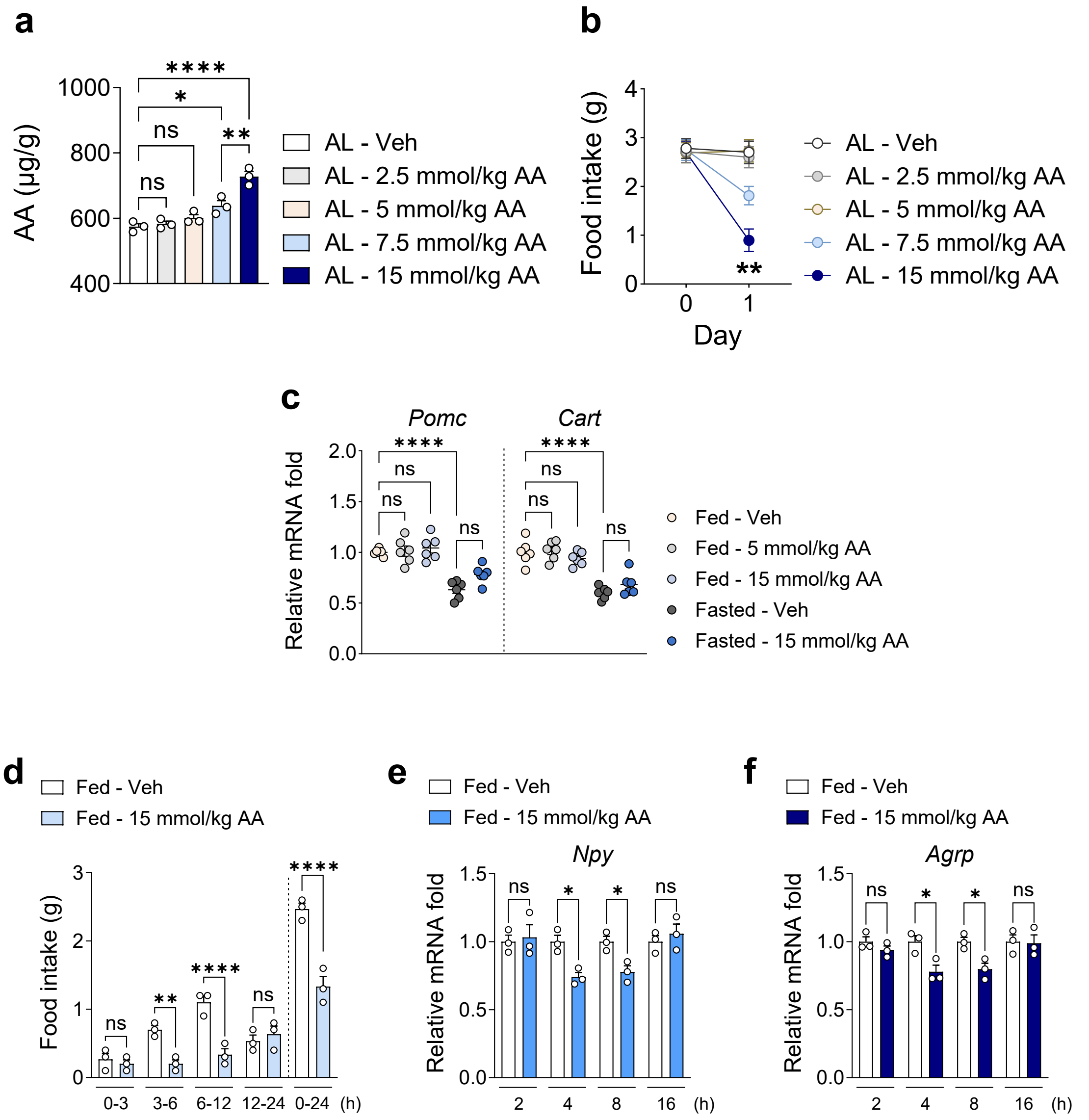

Supplementary Figure 4. Hypothalamic delivery of AA leads to reductions in food intake and orexigenic neuropeptide expression in male DIO mice. a, b DIO mice received a single i.p. administration of 2.5, 5, 7.5, or 15 mmol/kg AA under AL feeding (n=3); changes in **(a)** AA levels in the hypothalamus measured by LC-MS/MS, and **(b)** food intake measured daily. **c** Relative mRNA levels of hypothalamic anorexigenic neuropeptides. RNA was isolated from the hypothalamus of DIO mice harvested 4 h after a single i.p. injection of AA (5 or 15 mmol/kg) under fed or 24-h fasted conditions (n=6). **d–f** DIO mice received a single i.p. administration of 15 mmol/kg AA under AL feeding (n=3); changes in **(d)** food intake, and relative mRNA levels of **(e)** *Npy*, and **(f)** *Agrp*. Statistical significance was determined by one-way ANOVA followed by a *post hoc* Tukey test in panel **a, b**, and two-way ANOVA followed by a *post hoc* Tukey test in panels **c–f**. $^{*}P < 0.05$, $^{**}P < 0.01$, $^{****}P < 0.0001$; ns, no significance. Data are mean ± SEM.

Supplementary Figure 5

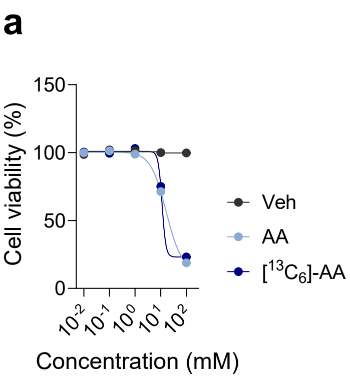

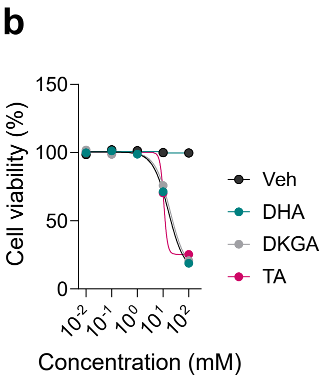

Supplementary Figure 5. Effects of AA and its metabolites on viability in N41 cells. a, b Cells were treated with 0.01–100 mM of **(a)** AA, [$^{13}C_6$]-AA, or **(b)** DHA, DKGA, TA for 24 h (n=6). Data are mean ± SEM.

Supplementary Figure 6

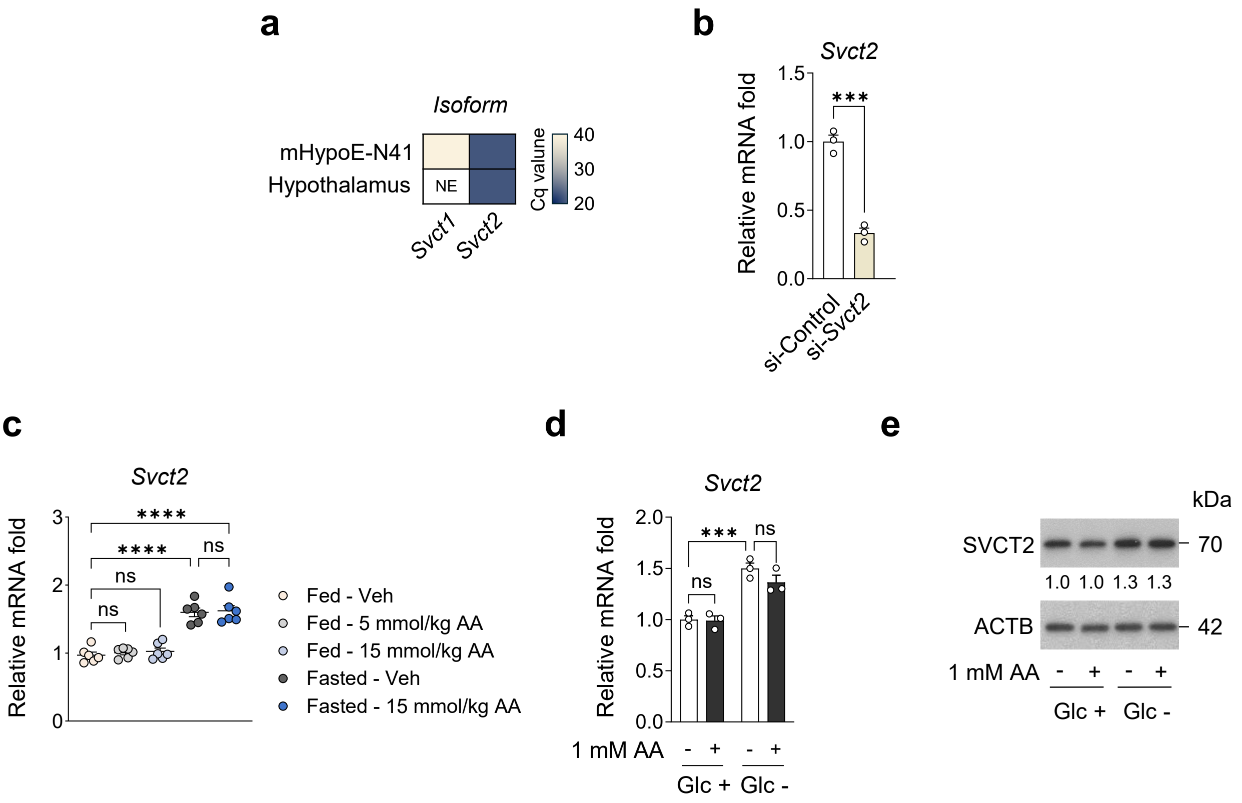

Supplementary Figure 6. Fasting upregulates SVCT2 expression in the hypothalamus of DIO mice and in N41 cells. a Expression of *Svct* in N41 cells and hypothalamic tissue (n=3). **b** Efficiency of *Svct2* knockdown after transfection of N41 cells with siRNA (n=3). **c** Relative mRNA level of *Svct2* in the hypothalamus of male mice under fed or 24-h fasted conditions. RNA was isolated from the hypothalamus harvested 4 h after a single i.p. injection of 15 mmol/kg AA (n=6). **d, e** N41 cells were treated with 1 mM AA for 4 h under normal (Glu +) or glucose-deprived conditions (Glu –) (n=3): **(d)** relative mRNA levels of *Svct2* and **(e)** western blot analysis of SVCT2 protein. Blot quantifications represent the mean values obtained from 3 biological replicates. Statistical significance was determined by two-tailed unpaired Student's *t*-test in panel **b** and two-way ANOVA followed by a *post hoc* Tukey test in panels **c**–**e**. ****P* < 0.001, *****P* < 0.0001; ns, no significance; ne, not expressed. Data are mean ± SEM.

Supplementary Figure 7

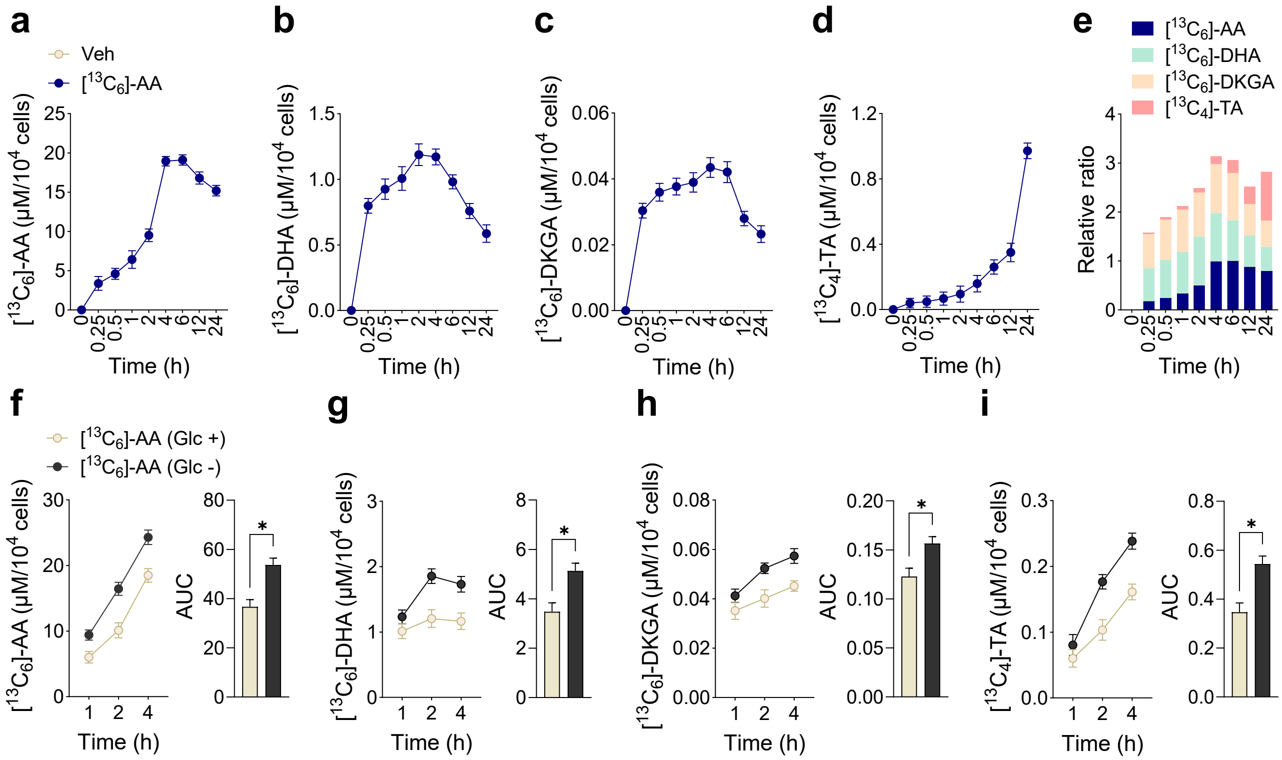

Supplementary Figure 7. Metabolic fate of AA under normal or glucose-deprived conditions in N41 cells. a–e Metabolic flow of ^{13}C-labeled AA after treatment with 1 mM [$^{13}C_6$]-AA for up to 24 h (n=3); **(a)** [$^{13}C_6$]-AA, **(b)** [$^{13}C_6$]-DHA, **(c)** [$^{13}C_6$]-DKGA, **(d)** [$^{13}C_4$]-TA, and **(e)** relative ratios of ^{13}C-labeled AA and its metabolites. **f–i** Metabolic flow of ^{13}C-labeled AA after treatment with 1 mM ^{13}C-labeled AA under normal (Glu +) or glucose-deprived conditions (Glu –) for up to 4 h (n=3); **(f)** [$^{13}C_6$]-AA, **(g)** [$^{13}C_6$]-DHA, **(h)** [$^{13}C_6$]-DKGA, and **(i)** [$^{13}C_4$]-TA. Statistical significance was determined by two-tailed unpaired Student's *t*-test. *$P < 0.05$; AUC, area under the curve. Data are mean ± SEM.

Supplementary Figure 8

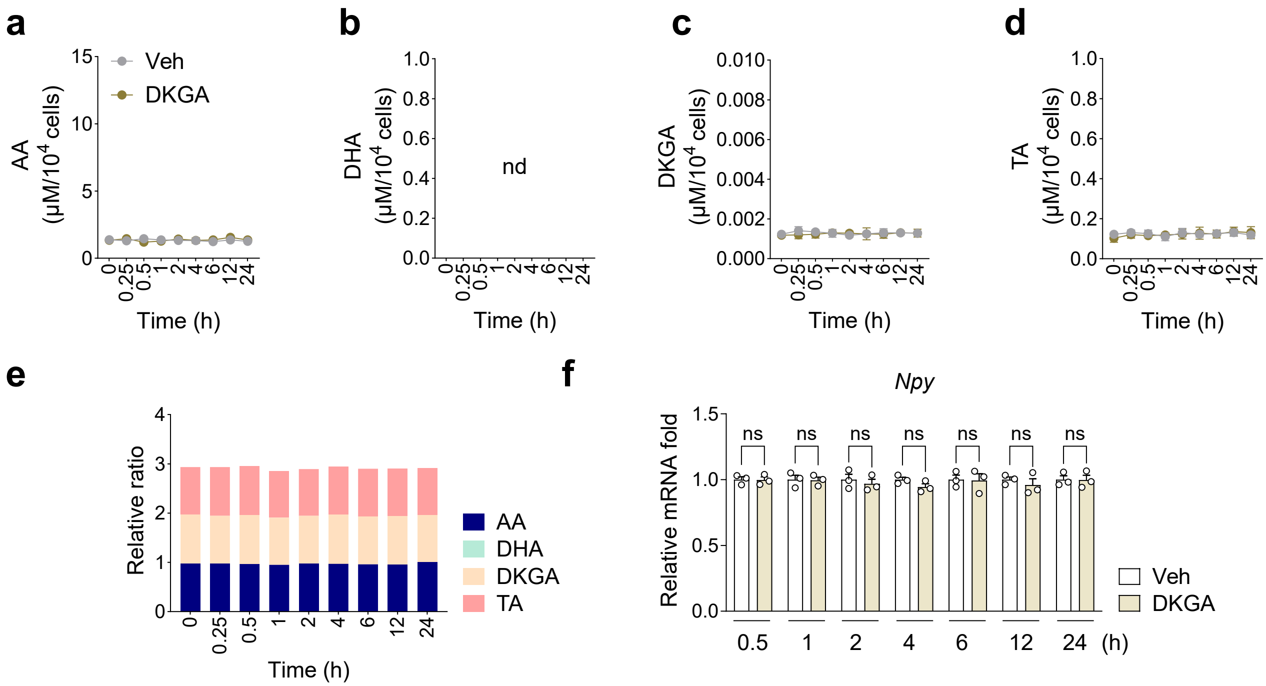

Supplementary Figure 8. DKGA treatment does not alter AA metabolite levels or *Npy* expression in N41 cells. a–e Metabolic flow of DKGA (n=3): **(a)** AA, **(b)** DHA, **(c)** DKGA, **(d)** TA, and **(e)** relative ratio of AA metabolites. **f** Relative mRNA levels of *Npy* upon DKGA treatment up to 24 h (n=3). Statistical significance was determined by two-way ANOVA followed by a *post hoc* Tukey test. ns, no significance; nd, not detected. Data are mean ± SEM.

Supplementary Figure 9

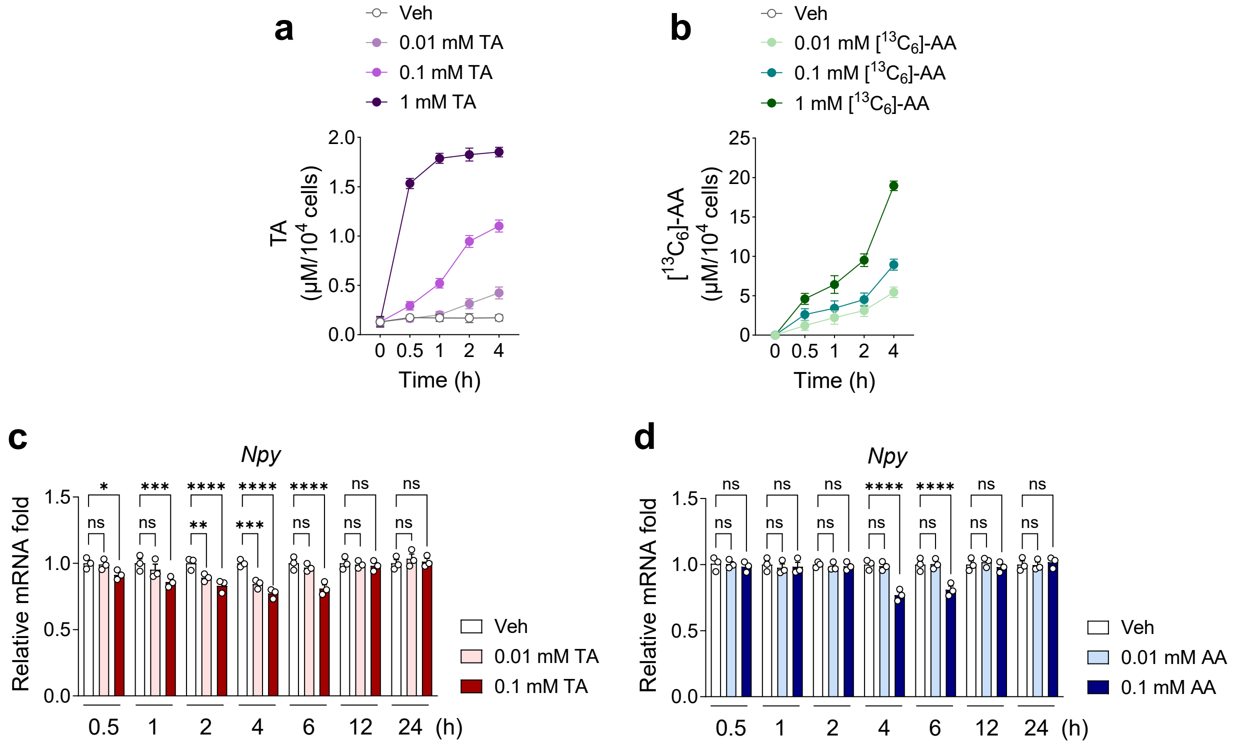

Supplementary Figure 9. TA downregulates *Npy* expression at lower doses and within a shorter time than does AA in N41 cells. a Intracellular TA levels upon treatment with 0.01, 0.1, or 1 mM TA (n=3). **b** Intracellular [$^{13}C_6$]-AA levels upon treatment with 0.01, 0.1, or 1 mM [$^{13}C_6$]-AA (n=3). **c, d** Relative mRNA levels of *Npy* upon treatment with **(c)** 0.01 or 0.1 mM TA, or **(d)** 0.01 or 0.1 mM AA (n=3). Statistical significance was determined by two-way ANOVA followed by a post hoc Tukey test. *P < 0.05, **P < 0.01, ***P < 0.001, ****P < 0.0001. ns, no significance. Data are mean ± SEM.

Supplementary Figure 10

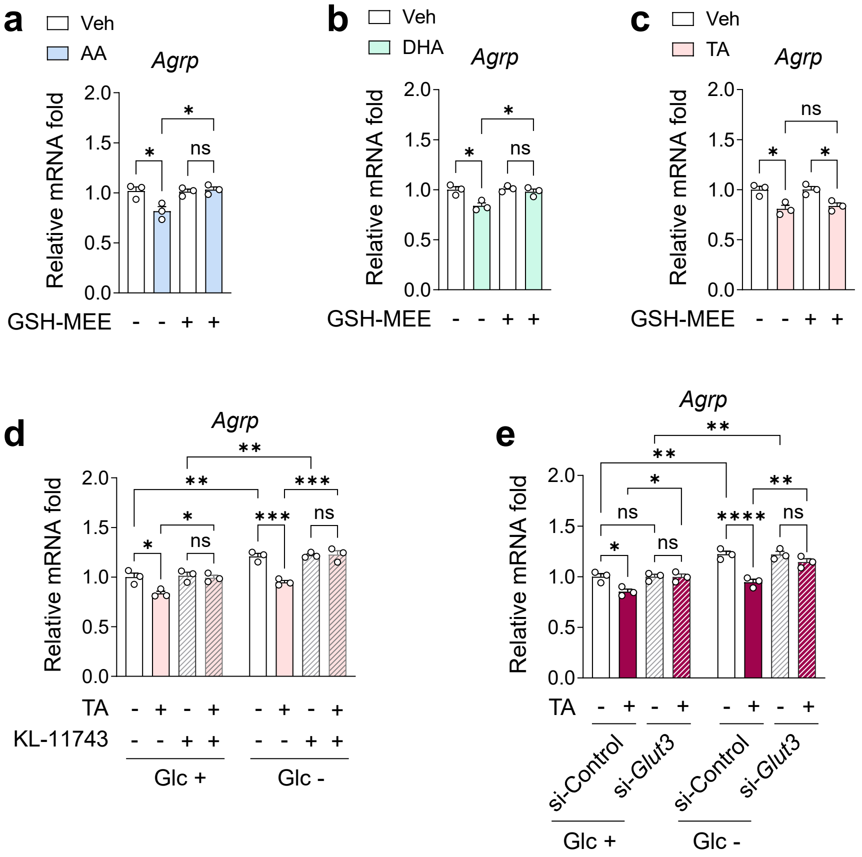

Supplementary Figure 10. TA also downregulates *Agrp* expression via GLUT3 in N41 cells. a–**c** Relative mRNA levels of *Agrp* in the presence or absence of an intracellular reducing agent (1 mM GSH-MEE, pre-treatment for 12 h), following treatment with 1 mM **(a)** AA, **(b)** DHA, or **(c)** TA for 4 h (n=3). **d** Relative mRNA levels of *Agrp* in the presence or absence of a GLUT inhibitor (0.1 mM KL-11743, pre-treatment for 1 h), following treatment with 0.1 mM TA for 1 h under normal (Glu +) or glucose deprived conditions (Glu –) (n=3). **e** Relative mRNA levels of *Agrp* after GLUT3 knockdown via siRNA-mediated gene silencing, followed by treatment with 0.1 mM TA for 1 h under normal (Glu +) or glucose deprived conditions (Glu –) (n=3). Statistical significance was determined by two-way ANOVA followed by a *post hoc* Tukey test. *P < 0.05, **P < 0.01, ***P < 0.001, ****P < 0.0001. ns, no significance. Data are mean ± SEM.

Supplementary Figure 11

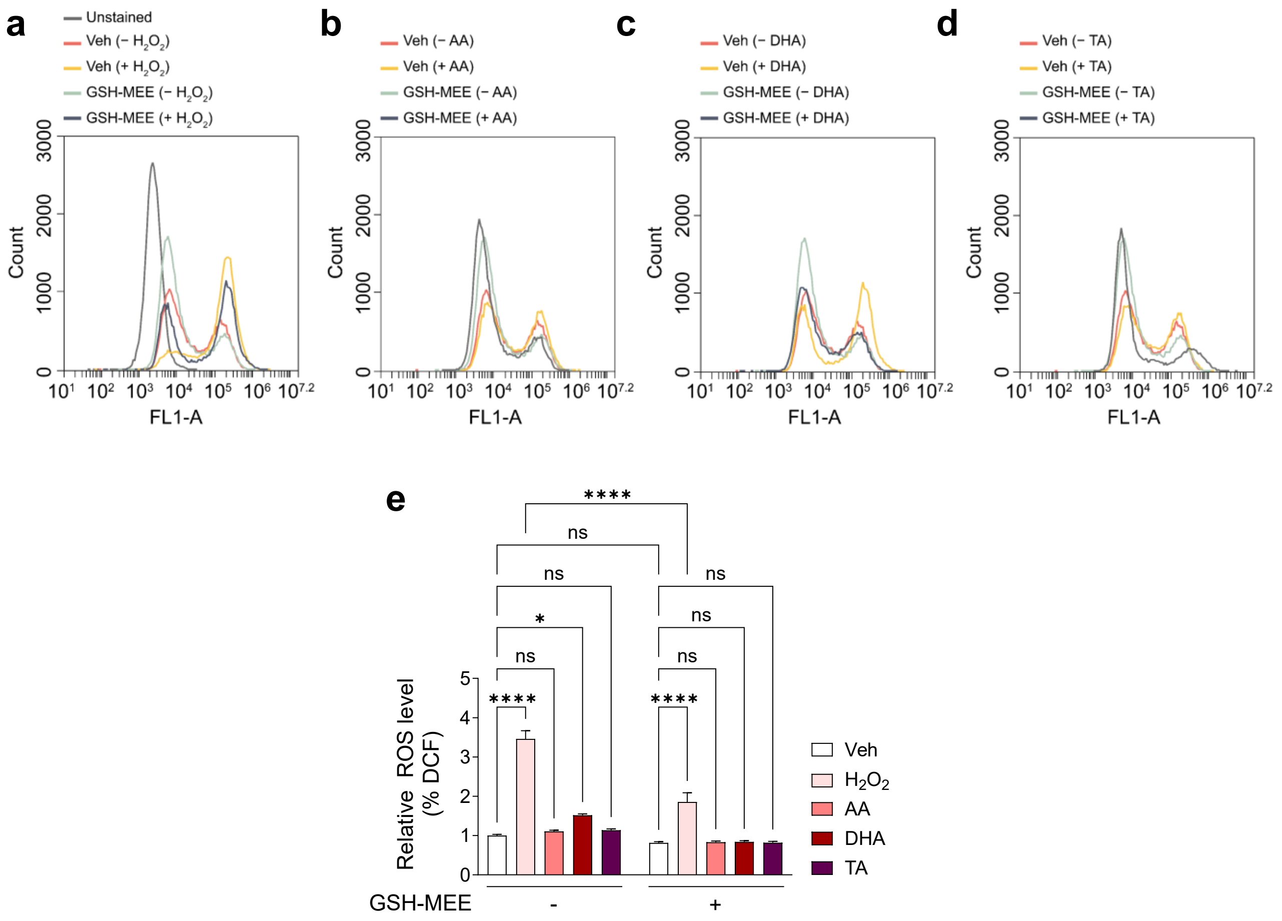

Supplementary Figure 11. Intracellular ROS levels in N41 cells measured using the DCFDA assay. Cells were treated with 1 mM H_2O_2, AA, DHA, or TA for 4 h in the presence or absence of GSH-MEE. ROS-sensitive fluorometric probe DCFDA was used for flow cytometry. **a**–**d** Histogram peaks for DCFDA (FL1). **e** Relative ROS levels (n=3). Statistical significance was determined by two-way ANOVA followed by a *post hoc* Tukey test. *P < 0.05, ****P < 0.0001; ns, no significance. Data are mean ± SEM.

Supplementary Figure 12

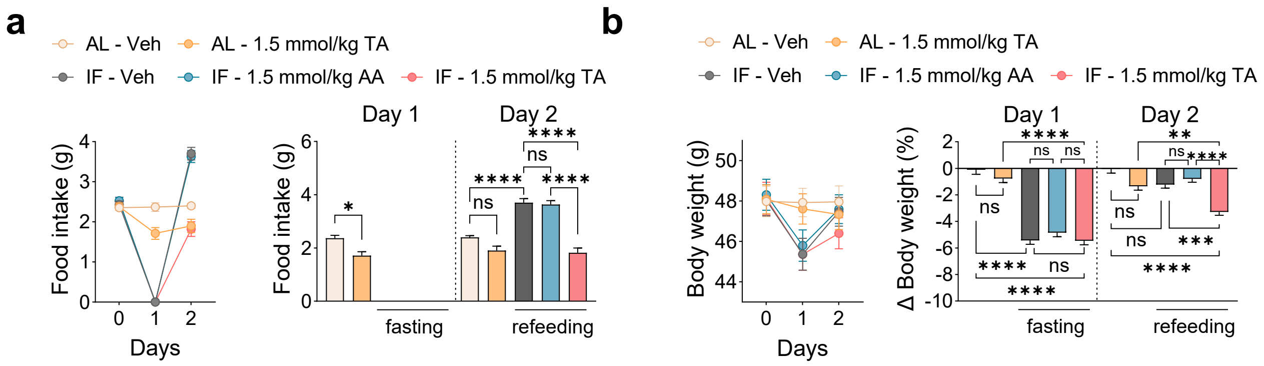

Supplementary Figure 12. Acute effects of TA administration on food intake and body weight under either AL or IF in male DIO mice. a, b DIO mice were subjected to either AL or IF regimen for 50 days, with daily i.p. injections of Veh, 1.5 mmol/kg TA, or 1.5 mmol/kg AA. On days 0, 1, and 2, changes in **(a)** daily food intake and **(b)** body weight (n=6). Statistical significance was determined by two-way ANOVA followed by a *post hoc* Tukey test. *$P < 0.05$, **$P < 0.01$, ***$P < 0.001$, ****$P < 0.0001$; ns, no significance. Data are mean ± SEM.

Supplementary Figure 13

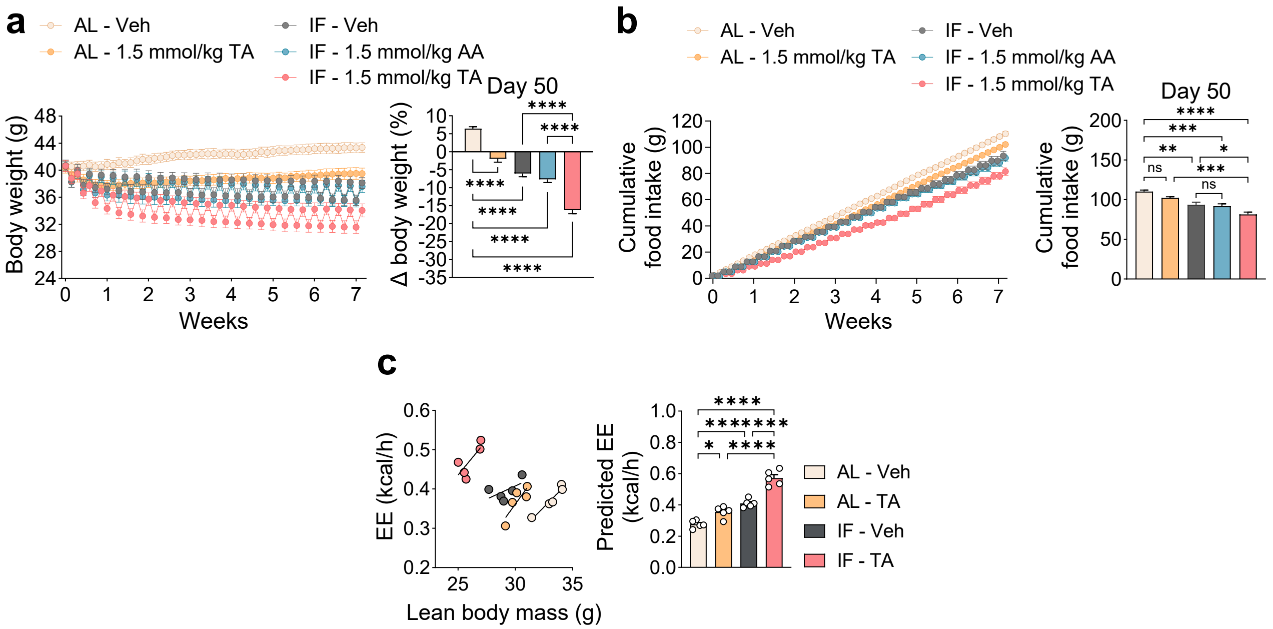

Supplementary Figure 13. Administration of TA in combination with IF enhances anti-obesity effects in female DIO mice. a–c Changes in **(a)** body weight measured daily (left panel) and on day 50 (relative to day 0, right panel), **(b)** cumulative food intake measured daily (left panel) and on day 50 (relative to day 0, right panel), and **(c)** regression-based analysis of energy expenditure (EE) against lean body mass (left panel) and EE values adjusted for lean body mass using ANCOVA (right panel) in mice injected intraperitoneally with 1.5 mmol/kg TA (n=5). Statistical significance was determined by two-way ANOVA followed by a *post hoc* Tukey test. *$P < 0.05$, **$P < 0.01$, ***$P < 0.001$, ****$P < 0.0001$; ns, no significance. Data are mean ± SEM.

Supplementary Figure 14

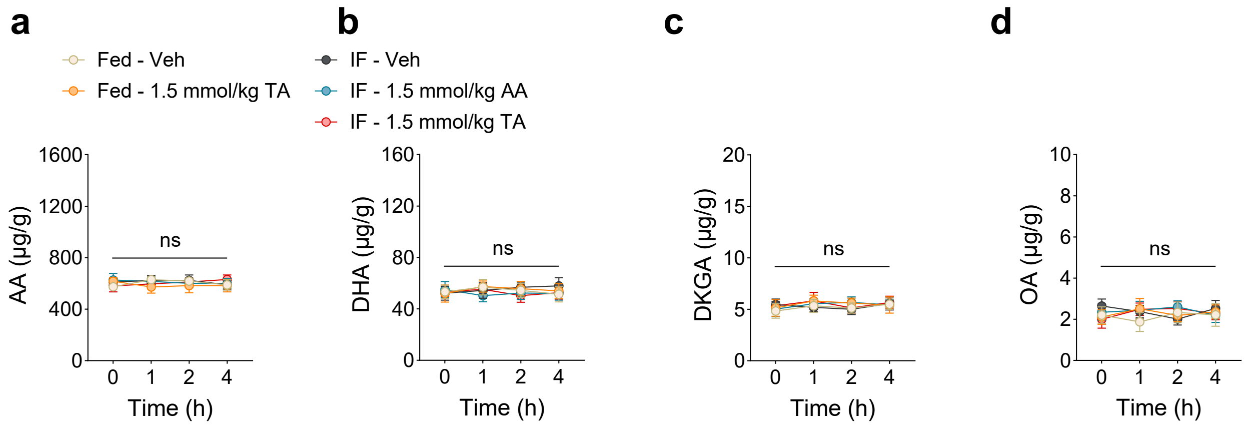

Supplementary Figure 14. TA combined with IF does not alter the levels of AA, DHA, DKGA, or OA in the hypothalamus of DIO mice. a–d Metabolic flow of TA or AA in the hypothalamus of male mice after a single i.p. injection of 1.5 mmol/kg TA (under fed or 24-h fasted conditions) or 1.5 mmol/kg AA (under 24-h fasted conditions only) (n=3); **(a)** AA, **(b)** DHA, **(c)** DKGA, and **(d)** OA. Statistical significance was determined by two-way ANOVA followed by a *post hoc* Tukey test. ns, no significance. Data are mean ± SEM.

Supplementary Figure 15

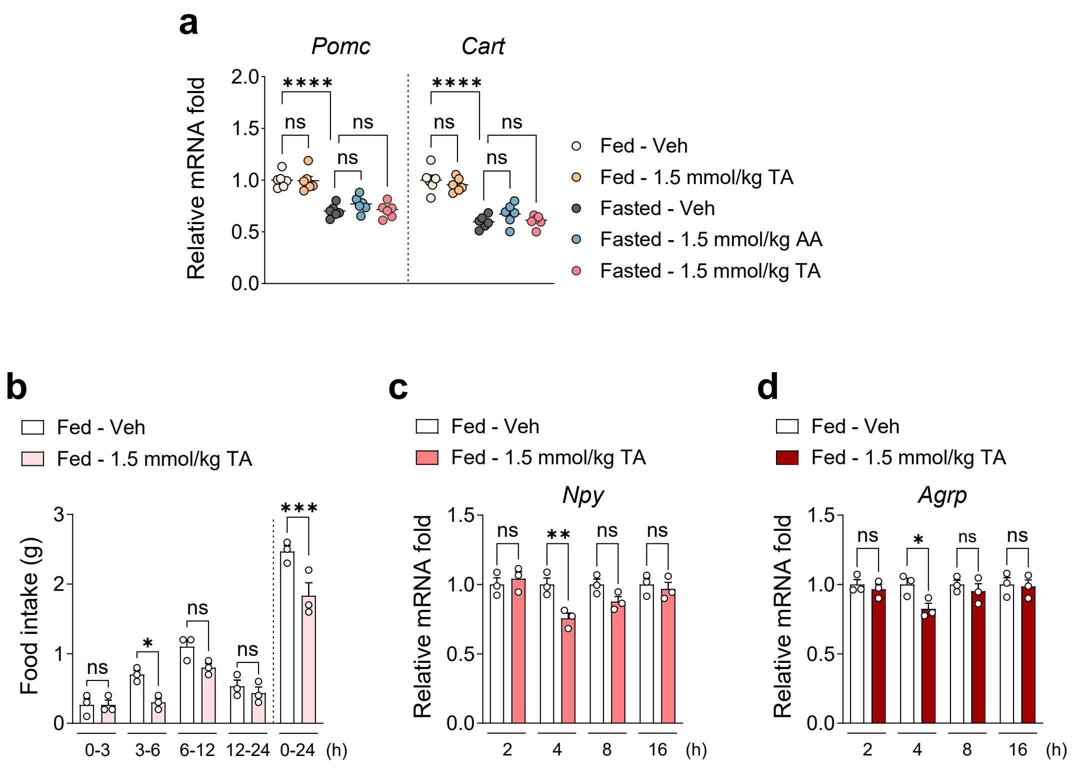

Supplementary Figure 15. Effects of a single administration of TA on hypothalamic neuropeptide expression and feeding behavior in male DIO mice. **a** Relative mRNA levels of hypothalamic anorexigenic neuropeptides. RNA was isolated from the hypothalamus of mice harvested 4 h after a single i.p. injection of 1.5 mmol/kg TA under fed or 24-h fasted conditions (n=5). **b–d** Mice received a single i.p. administration of 1.5 mmol/kg TA under AL feeding (n=3); changes in **(b)** food intake, and relative mRNA levels of **(c)** *Npy*, and **(d)** *Agrp*. Statistical significance was determined by two-way ANOVA followed by a *post hoc* Tukey test. $^*P < 0.05$, $^{**}P < 0.01$, $^{***}P < 0.001$, $^{****}P < 0.0001$; ns, no significance. Data are mean ± SEM.

Supplementary Figure 16

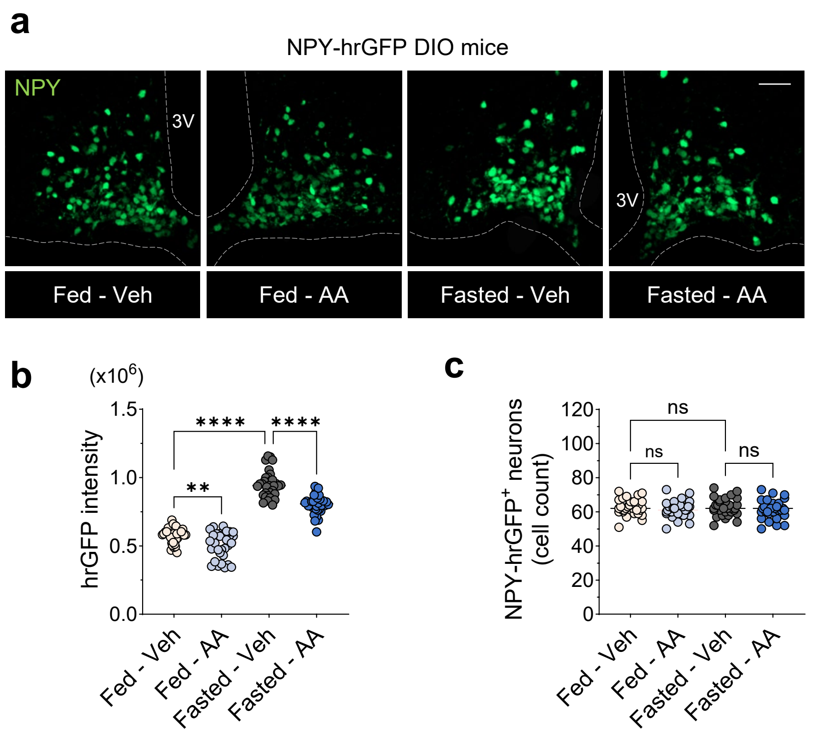

Supplementary Figure 16. AA combined with IF leads to a more pronounced reduction of hrGFP fluorescence intensity in NPY-hrGFP mice. a–c Immunohistochemistry analysis of NPY fluorescence intensity in the ARC of **(a)** NPY-hrGFP DIO mice. Mice fed an HFD for 16 weeks received a single i.p. injection of 15 mmol/kg AA, and brains were harvested 4 h later. Eight brain slices (35 μm thickness) were obtained from each of 4 mice per group (n=32) to determine the intensity of **(b)** hrGFP, and **(c)** the number of hrGFP-positive cells. Scale bars, 20 μm. Statistical significance was determined by two-way ANOVA followed by a *post hoc* Tukey test. $^{**}P < 0.01$, $^{****}P < 0.0001$. ns, no significance. Data are mean ± SEM.

Supplementary Table 1. List of antibodies

Antibody	Manufacturer	Dilution
SVCT2	Novus Biologicals (NBP2-13319)	1:1000
GLUT3	Thermo Fisher (MA5-32697)	1:5000 for WB<br>1:100 for IHC
ACTB	Santa Cruz Biotechnology (sc-47778)	1:10000
Donkey anti-mouse IgG Alexa Fluor 647	Jackson ImmunoResearch (715-605-151)	1:500
Donkey anti-goat IgG Alexa Fluor 647	Jackson ImmunoResearch (705-605-147)	1:500
Donkey anti-rabbit IgG Cy3	Jackson ImmunoResearch (711-165-152)	1:500

Supplementary Table 2. Sequences of primers used for qRT-PCR

Gene	Forward primer (5′ to 3′)	Reverse primer (5′ to 3′)
Npy	CAGAAAACGCCCCCAGAA	AAAAGTCGGGAGAACAAGTTTCATT
Agrp	CTGCAGACCGAGCAGAAGA	TGCGACTACAGAGGTTCGTG
Pomc	GAACAGCCCCTGACTGAAAA	ACGTTGGGGTACACCTTCAC
Cart	CGAGAAGAAGTACGGCCAAGTCC	GGAATATGGGAACCGAAGGTGG
Svct1	CTCTCATCCAGACTACAGTGGG	GGTGTTGAGAGGCATACTCCAG
Svct2	GGACGGCATACAAGTTCCAGCT	AGCCATAGTCGGTGCTGTTGGA
Glut3	CCGCTTCTCATCTCCATTGTCC	CCTGCTCCAATCGTGGCATAGA
Actb	CATTGCTGACAGGATGCAGAAGG	TGCTGGAAGGTGGACAGTGAGG

Supplementary Table 3. Precursor-to-fragment ion transitions in LC-MS/MS analysis

Analyte	Precursor ion (m/z)	Fragment ion (m/z)	Collision energy (eV)
AA	175.0	115.0	8
[$^{13}C_6$]-AA	181.0	119.0	8
DHA	173.0	142.9	6
[$^{13}C_6$]-DHA	179.0	147.9	6
DKGA	191.0	146.9	4
[$^{13}C_6$]-DKGA	197.0	151.9	4
OA	89.0	60.9	6
TA	135.0	74.9	10
[$^{13}C_4$]-TA	139.0	76.9	10
Glucose	178.9	58.9	16
[$^{13}C_2$]-Glucose	180.9	58.0	12
